# Attribute-based encryption scheme with multi-keyword search and supporting attribute revocation in cloud storage

**DOI:** 10.1371/journal.pone.0205675

**Published:** 2018-10-12

**Authors:** Shangping Wang, Lisha Yao, Yaling Zhang

**Affiliations:** 1 School of Science, Xi’an University of Technology, Xi’an, Shaanxi, China; 2 School of Computer Science and Engineering, Xi’an University of Technology, Xi’an, Shaanxi, China; King Saud University, SAUDI ARABIA

## Abstract

We propose an attribute-based encryption scheme with multi-keyword search and supporting attribute revocation in cloud storage environment, in which binary attributes and AND-gate access policy are used. Our proposal enjoys several advantages. Firstly, multi-keyword search is available, and only when a data user's attribute set satisfies access policy in keyword index, and keyword token generated by data user matches index successfully, then data user can obtain ciphertext containing keywords. In this way, more accurate keyword search is achievable. Secondly, the search privacy of data user is protected owing to cloud servers cannot obtain any knowledge of keywords which data user is interested in. Meanwhile, the ciphertext is able to be decrypted when data user's attribute set satisfies access policy specified in the ciphertext, which can both improve security of encryption and achieve secure fine-grained access control. Thirdly, the proposed scheme supports attribute revocation, in our scheme when a data user's attribute is revoked, the version number of attribute, non-revoked data users' secret keys and related ciphertexts will be updated, such that data user whose attribute is revoked does not decrypt updated ciphertext anymore. In addition, based on the assumption of decisional linear (DL) and decisional Diffie-Hellman (DDH), our scheme is proved to be secure against selectively chosen-keyword attacks and selectively chosen-plaintext attacks respectively, and it also ensures token privacy security.

## Introduction

With the fast development of information technology, cloud storage now plays a very crucial role [1] in our daily life. For the sake of insuring data security, the important data that are uploaded to cloud server needs to be kept confidential, which requires data owners to encrypt private files before uploading. Meanwhile, it is also necessary to quickly find required files for data users by keyword searching from a vast amount of encrypted data. Therefore, in order to enable a secure keyword search and protect data user's search privacy, setting the keyword index of file is essential. That means that, although cloud server provides a search service, it does not know any information of keyword searching by data users. Consequently, it has important theoretical value and practical significance to study secure and practical attribute-based encryption schemes that sustaining both attribute revocation and multi-keyword search.

In order to provide fine-grained access control for encrypted data, Sahai and Waters first proposed the notion of attribute-based encryption (ABE) in [2], which achieved one-to-many secure services based on public key encryption, and it ensured efficient encrypted access policy. Indeed, many attribute-based encryption (ABE) schemes have been presented, Goyal et al. [3] further defined the concept of attribute-based encryption (ABE). In general, attribute-based encryption (ABE) schemes are classified into two categories: One kind is key-policy attribute-based encryption (KP-ABE) [4–6], in which data user's secret key and ciphertext are relevant to access policy and attribute set, respectively. The other kind is ciphertext-policy attribute-based encryption (CP-ABE), which was first put forward by Bethencourt et al. in [7] that was proved to be safe under the general group model. In a CP-ABE scheme, the data user's secret key is related to attribute set and ciphertext is related to specific access policy. In 2008, Goyal et al. [8] proposed a CP-ABE scheme that was secure under the decisional bilinear Diffie-Hellman (DBDH) assumption. In 2012, cheng et al. [9] presented a CP-ABE scheme in large universe set, which introduced attribute union, and it reduced storage and computational overhead of existing CP-ABE schemes. In 2016, Li et al. [10] added a testing phase to avoid unnecessary operation in their scheme and the proposal was proved to be safe under the decisional Diffie-Hellman (DDH) assumption. For resource-constrained devices, Odelu et al. [11] proposed a scheme that had constant size ciphertexts and secret keys. Recently, Shynu et al. [12] presented a notion of attributes hierarchical, constructed a separate database system for a meaningful data user's attribute set, and it solved the problem of attributes management.

Keyword searchable encryption is an effective solution to quickly find desired files from a vast amount of encrypted data managed in cloud servers. In 2003, Dan et al. [13] proposed a public key encryption with keyword search (PEKS) scheme. In 2010, Li et al. [14] presented a fuzzy keyword search for encrypted data scheme, which enhanced system usability by approximate matching of files and keywords. Subsequently, identity-based public key encryption and keyword searchable schemes were proposed in [15]. In 2010, Kamara et al. [16] put forward three models for data encryption and search. Since most of these schemes cannot support multi-keyword search, to address this problem, Cao et al. [17] proposed an encryption and multi- keyword sequence search scheme, which allowed multiple keywords in search phase, and it returned documents in relevant order. In 2012, the public key encryption and multi-keyword search scheme was proposed in [18]. In 2016, Miao et al. [19] presented an attribute-based multi-keyword search encryption scheme for personal health records in multi-owner environment, which provided an application direction for multi-keyword searchable encryption. In 2017, Huang et al. [20] proposed a multi-sever multi-keyword searchable encryption scheme, which is proved to be secure against adaptive chosen keyword attack.

From a few years ago to today, many CP-ABE schemes that were sustaining attribute revocation have been mentioned. In 2010, Yu et al. [21] implemented a direct revocation of attributes in virtue of an agent, in which proxy key can be used to generate proxy re-encrypted ciphertext, and the scheme also had a capability of updating all corresponding secret keys of each legitimate data users. Afterwards, Yang et al. [22] presented a CP-ABE scheme that were supporting attribute revocation, in which attribute authority was responsible for updating ciphertexts and non-revoked data users' corresponding secret keys related to revoked attribute. In 2014, Xiong et al. [23] put forward a CP-ABE scheme that supporting universe attribute revocation, which was built on multiple minimum attribute sets of sharing re-encryption keys. When a universe attribute needs to be revoked, the cloud server performs the operation of re-encrypted ciphertext. In 2016, according to single authority attribute-based encryption (ABE) schemes, Chow [24] presented a scheme that was attribute-based encryption (ABE) with supporting multi-authority and revocation. In 2017, Liu et al. [25] proposed an attribute-based encryption scheme that was sustaining both outsourced decryption algorithm and attribute revocation, which set a randomized version number for each attribute, thus attribute revocation is effectively implemented.

Recently, Wang et al. [26] proposed a CP-ABE scheme, which supported keyword searchable and attribute update in cloud storage. Our solution and scheme [26] are different in the following aspects: Firstly, access policy is different. The scheme [26] adopts linear secret sharing (LSSS) in the specific algorithm design, while our solution uses AND-gate access policy. Secondly, attribute update and revocation are different. Attribute update is used in [26], which updates a data user's original attribute to a new attribute and also updates the data user's secret key associated with the attribute. Our scheme is attribute revocation. Although scheme [26] also involves attribute revocation, it is still different from ours. We set the version number for each attribute, when the version number of revocation attribute changes, related ciphertexts and all non-revoked data users' secret keys are updated. Thirdly, the security proof method of schemes is different. The scheme [26] proves that the algorithms resist chosen-keyword attack based on the hard problem of bilinear Diffie-Hellman (BDH), and the scheme is proved to be secure against chosen-plaintext attack under the general bilinear group model. However, the security proof of our proposal is based on the hard problems. According to the assumption of decisional linear (DL) and decisional Diffie-Hellman (DDH), our scheme is proved to be secure against selectively chosen-keyword attack and selectively chosen-plaintext attack, respectively. At the same time, our solution is proved to enjoy token privacy security by using the unidirectional and collision-resistance of hash function. In addition, our scheme analyzes the forward and backward security for attribute revocation.

### 1.1 Our contributions

Considering that most of existing CP-ABE schemes cannot support attribute revocation and multi-keyword search, we present a CP-ABE scheme with multi-keyword search and supporting attribute revocation in cloud storage. The innovations can be summarized as follows:

1. Our scheme supports attribute revocation, when a data user's attribute is revoked, the version number of attribute, non-revoked data users' secret keys and related ciphertexts will be updated, such that data user whose attribute is revoked does not decrypt updated ciphertext anymore.
2. After data owners upload overall ciphertext of encrypted data to cloud server, the keyword search is used to quickly find required file. Compared to a single keyword search, the multi-keyword search is closer to real application. For the consideration of this issue, our scheme supports multi-keyword search.

## Preliminaries

### 2.1 Bilinear map[27]

Let $G,G_{T}$ be two multiplicative cyclic groups with prime order *p*, *g* be a generator of group G. Let $e:G\times G\to G_{T}$ be a bilinear map with following properties:

1. Bilinearity: For any $a,b\in {\mathbb{Z}}_{p}$, exist *e*(*g*^*a*^,*g*^*b*^) = *e*(*g*,*g*)^*ab*^.
2. Non-degeneracy: For g∈G, such that *e*(*g*,*g*) ≠ 1.
3. Computability: For any u,v∈G, *e*(*u*,*v*) is efficiently computed.

### 2.2 Access policy[28]

We denote by *U* = {*attr*_1_,*attr*_2_,⋯,*attr*_*n*_} the universe set of attributes, where *n* is the size of *U*, namely |*U*| = *n*. Let *Attr* be attribute set of a data user. We introduce an n-bit string ${\hat{v}}_{1}{\hat{v}}_{2}\cdots {\hat{v}}_{n}$ to express data user's attribute set $Attr={\hat{v}}_{1}{\hat{v}}_{2}\cdots {\hat{v}}_{n}$ as follows:

$$\left\{ \begin{array}{l} {\hat{v}}_{j}=v_{j}:attr_{j}\in Attr\\ {\hat{v}}_{j}={}^{\neg }v_{j}:attr_{j}\notin Attr \end{array}\right.\;j\in \left[1,n\right]$$

For example, let *n* = 5, suppose a data user's attribute set is {*attr*_1_,*attr*_3_,*attr*_5_}, then it may be expressed as *Attr* = *v*_1_^¬^*v*_2_*v*_3_^¬^*v*_4_*v*_5_.

We adopt AND-gate access policy and introduce an n-bit string ${\tilde{v}}_{1}{\tilde{v}}_{2}\cdots {\tilde{v}}_{n}$ to express AND-gate access policy $S={\tilde{v}}_{1}{\tilde{v}}_{2}\cdots {\tilde{v}}_{n}$ as follows:

$$\left\{ \begin{array}{l} {\tilde{v}}_{j}=v_{j}:attr_{j}\in S\\ {\tilde{v}}_{j}={}^{\neg }v_{j}:attr_{j}\notin S \end{array}\right.\;j\in \left[1,n\right]$$

For example, let *n* = 5, suppose the access policy is {*attr*_1_,*attr*_5_}, then it may be expressed as *S* = *v*_1_^¬^*v*_2_^¬^*v*_3_^¬^*v*_4_*v*_5_.

For a data user's attribute set $Attr={\hat{v}}_{1}{\hat{v}}_{2}\cdots {\hat{v}}_{n}$ and an access policy $S={\tilde{v}}_{1}{\tilde{v}}_{2}\cdots {\tilde{v}}_{n}$. If for all *j* ∈ [1,*n*], we have ${\hat{v}}_{j}\in S$, that is ${\hat{v}}_{j}={\tilde{v}}_{j}$ (${\hat{v}}_{j}$ represents the value of *j*-th attribute in data user's attribute set *Attr*, ${\tilde{v}}_{j}$ represents the value of *j*-th attribute in access policy *S*), we can say that the attribute set *Attr* of data user satisfies access policy *S*. For convenience, we define a function *γ*(*Attr*,*S*) ∈ {0,1}, when *γ*(*Attr*,*S*) = 0, it indicates that data user's attribute set *Attr* does not satisfy access policy *S*. When *γ*(*Attr*,*S*) = 1, it indicates that the attribute set *Attr* of data user satisfies access structure *S*.

### 2.3 Complexity assumption

In our proposal, the security depends on Decisional linear (DL) assumption [29] and Decisional Diffie-Hellman (DDH) assumption [30]. The specific description is:

**Definition 1 (Decisional linear assumption).** If for all polynomial-time adversary A who could successfully distinguish tuple $\left(g,f,h,f^{r_{1}},g^{r_{2}},h^{r_{1}+r_{2}}\right)$ from tuple $\left(g,f,h,f^{r_{1}},g^{r_{2}},R\right)$ with a negligible advantage, and the advantage ADV(A) of polynomial-time adversary A can be marked as

$$ADV\left(A\right)=\left|\mathrm{Pr}\left[A\left(g,f,h,f^{r_{1}},g^{r_{2}},h^{r_{1}+r_{2}}\right)=1\right]-\mathrm{Pr}\left[A\left(g,f,h,f^{r_{1}},g^{r_{2}},R\right)=1\right]\right|$$

where g,f,h,R∈G, $r_{1},r_{2}\in {\mathbb{Z}}_{p}^{*}$.

**Definition 2 (Decisional Diffie-Hellman assumption).** If for all polynomial-time adversary A who could successfully distinguish tuple $\left(g,g^{z_{1}},g^{z_{2}},g^{z_{1}z_{2}}\right)$ from tuple $\left(g,g^{z_{1}},g^{z_{2}},Q\right)$ with a negligible advantage, and the advantage ADV(A) of polynomial-time adversary A can be marked as

$$ADV\left(A\right)=\left|\mathrm{Pr}\left[A\left(g,g^{z_{1}},g^{z_{2}},g^{z_{1}z_{2}}\right)=1\right]-\mathrm{Pr}\left[A\left(g,g^{z_{1}},g^{z_{2}},Q\right)=1\right]\right|$$

where g,Q∈G, $z_{1},z_{2}\in {\mathbb{Z}}_{p}^{*}$.

## Our scheme

### 3.1 System model

The section contains overall framework of our scheme and the construction of solution.

#### 3.1.1 System framework

**Fig 1:**
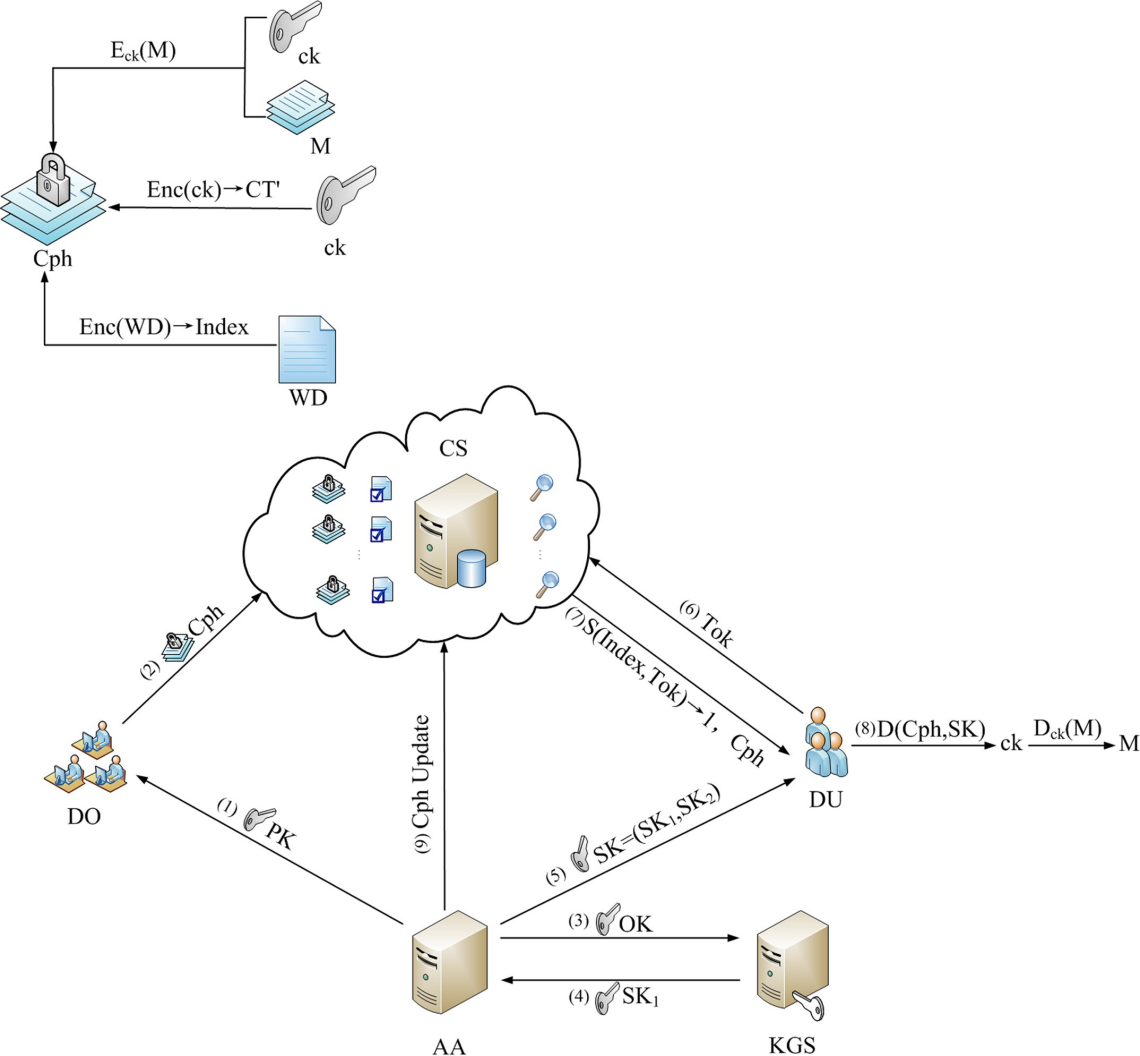
A framework of scheme.

Our scheme structure is shown in Fig 1. It contains following five entities:

**Attribute Authority (AA):** The AA is attribute authority, which is responsible for system's initial establishment and the local secret key generation of data user. Simultaneously, it distributes corresponding secret key according to attribute set for data user. When an attribute is revoked, AA generates an update key and completes partial secret key update.

**Cloud Server (CS):** The CS stores ciphertext which containing encrypted files and keyword indexes generated by data owners. Afterwards, when a data user tends to search ciphertext, CS completes a matching of data user's token and keyword index. If matching succeeds, it sends ciphertext to data user. Additionally, in attribute revocation phase, CS is responsible for updating ciphertext.

**Key Generation Server (KGS):** The KGS generates data user's partial secret key, namely outsourced secret key, which effectively reduces the computational burden of AA. Besides, KGS is responsible for completing the update of outsourced secret key when attribute revocation happens.

**Data Owner (DO):** The DO encrypts keyword set and file to be shared, uploads ciphertext to cloud server. Only attribute set of data user who wants to access data satisfies access structure in ciphertext, that is *γ*(*Attr*,*S*) = 1, the encrypted data will be shared with data user. To be specific, the encryption operation to be completed by DO includes: the keyword index generation, the file encryption, and the encryption of key for encrypted file, hence ciphertext consists of three parts.

**Data User (DU):** When data user's attribute set satisfies access structure in ciphertext, then data user DU is able to access encrypted data and recover original plaintext. Specifically, DU generates desired keyword token and sends to cloud server CS, the CS makes a matching between search token and keyword index, if matching succeeds, DU can download corresponding ciphertext. In other words, DU is responsible for generating keyword token which he is interested in and decrypting ciphertext.

In particular, *PK* is public parameter published by attribute authority. *Cph* is ciphertext encrypted by data owner, and it includes three parts: 1) The keyword set *WD* is encrypted to generate keyword index, namely *Index*; 2) The encryption key *ck* is encrypted to obtain ciphertext *CT*′; 3) The file *M* is symmetrically encrypted by using encryption key *ck* to gain *E*_*ck*_(*M*). At last, data owner uploads ciphertext *Cph* to cloud server. Hereafter, *OK* is used to denote intermediate key, which is generated by attribute authority according to attribute set of data user, and it is sent to key generation server. The key generation server calculates data user's partial secret key based on *OK*, namely outsourced secret key *SK*_1_, which is sent to attribute authority. Followed by, the attribute authority generates data user's local secret key *SK*_2_, and obtains data user's secret key *SK* = (*SK*_1_,*SK*_2_), which is forwarded to data user. *Tok* is used to denote token generated by data user based on desired keyword set, which is used to match with keyword index. The cloud server executes search algorithm, if token matches index successfully, the cloud server transmits stored ciphertext *Cph* to data user. Then data user first decrypts ciphertext *Cph* with secret key *SK* to gain encryption key *ck*, and then symmetric decrypts ciphertext with *ck* to obtain file *M*. In addition, when an attribute needs to be revoked, attribute authority sends instructions to cloud server to update ciphertext.

#### 3.1.2 Standard definitions of our scheme

Let *U* be a universe set of attributes, *S* be an access policy, *Attr* be a data user's attribute set. Attribute-based encryption scheme with multi-keyword search and supporting attribute revocation in cloud storage consists of 9 algorithms:

**Setup**(*U*,*l*)→*PK*,*MSK*: The setup algorithm is executed by attribute authority AA. It inputs universe set of attributes *U* and security parameter *l*, and outputs system public key *PK* and master secret key *MSK*.

**Encrypt**: The encryption algorithm is run by data owner DO, including the following two parts:

i) **Keyword-Encrypt**(*PK*,*S*,*WD*)→*Index*: This algorithm takes as inputs access policy *S*, public key *PK* and keyword set *WD*, and it then outputs the ciphertext *Index* of keyword set *WD*.
ii) **Encryption key-Encrypt**(*PK*,*S*,*ck*)→*CT*′: DO first symmetrically encrypts file *M* rely on encryption key *ck* to obtain *E*_*ck*_(*M*), and then encrypts encryption key *ck* as follows: This algorithm makes access policy *S*, public key *PK* and encryption key *ck* as input, it generates ciphertext *CT*′.

Finally, DO uploads overall ciphertext *Cph* = (*Index*,*E*_*ck*_(*M*),*CT*′) to cloud server CS.

**In-KeyGen**(*PK*,*MSK*,*Attr*)→*OK*: The intermediate key generation algorithm is executed by attribute authority AA. It makes public key *PK*, master secret key *MSK* and data user's attribute set *Attr* as input, then produces intermediate key *OK*, and sends it to key generation server KGS.

**Out-KeyGen**(*PK*,*OK*)→*SK*_1_: The outsourced secret key generation algorithm is executed by key generation server KGS. It takes public key *PK* and intermediate key *OK* as input, and outputs outsourced secret key *SK*_1_, then returns *SK*_1_ to attribute authority AA.

**KeyGen**(*PK*,*MSK*,*Attr*,*SK*_1_)→*SK*: The secret key generation algorithm is executed by attribute authority AA. It makes public key *PK*, master secret key *MSK*, data user's attribute set *Attr* and outsourced secret key *SK*_1_ as input, then generates data user's secret key *SK*, and transmits it to data user DO.

**TokenGen**(*PK*,*SK*_1_,*WD*′)→*Tok*: The token generation algorithm is executed by data user DU. It makes public key *PK*, outsourced secret key *SK*_1_ and desired keyword set *WD*′ as input, and it outputs token *Tok*, then forwards to cloud server CS.

**Search**(*Index*,*Tok*)→{0,1}: The search algorithm is executed by cloud server CS. It makes index and token as input, outputs 1 if index and token can match successfully, then cloud server CS sends ciphertext *Cph* to data user DU, otherwise outputs 0 and terminates.

**Decrypt**(*SK*_2_,*CT*′)→*ck*: The decryption algorithm is executed by data user DU. It makes local secret key *SK*_2_ and ciphertext *CT*′ of encryption key as input, generates encryption key *ck*, then it decrypts *E*_*ck*_(*M*) with *ck*, finally obtains file *M*.

**Attribute revocation**: The attribute revocation includes the following three aspects, note that only components related to revoked attribute will be updated.

(i) The attribute authority AA takes charge of generating update key: AA generates a new version number of revoked attribute according to its old version number, then obtains update key. This algorithm makes update key, public key *PK* and master secret key *MSK* as input, outputs updated public key and updated master secret key.
(ii) The non-revoked data user's secret key update: The attribute authority AA updates intermediate key *OK* and local secret key *SK*_2_ with update key, sends updated intermediate key to key generation server KGS. Then, KGS completes the update of outsourced secret key *SK*_1_, and transmits to AA. In the end, AA returns updated secret key to non-revoked data user.
(iii) The cloud server CS is in charge of updating ciphertext: The cloud server CS executes this algorithm. It makes update key and overall ciphertext as input, outputs updated keyword index and updated ciphertext of encryption key.

### 3.2 Security model

Under the cloud storage environment, we suppose that attribute authority and key generation server are all trusted. But cloud server is semi-trusted, such as it can execute protocols honestly but also attempt to gain extra information from the protocol.

#### 3.2.1 Selectively secure game for chosen-keyword attacks

**Setup:** First of all, adversary A sends a challenge access policy S* to challenger C, the challenger C runs **Setup** algorithm to generate public key *PK* and master secret key *MSK*, while keeping *MSK* secret.

**Phase 1:** Before Phase 1 begins, the challenger C initializes an empty keyword set query list *L*_*W*_, then adversary A issues the following polynomial times adaptive queries:

Outsourced secret key query: According to **In-KeyGen** algorithm and **Out-KeyGen** algorithm, the adversary A submits a query attribute set Attr* to challenger C. If attribute set Attr* does not satisfy access policy S*, the adversary A obtains outsourced secret key *SK*_1_, otherwise terminates.

Token query: The adversary A commits a query keyword set *WD*′. According to **TokenGen** algorithm, the challenger C inputs public key *PK*, outsourced secret key *SK*_1_ and query keyword set *WD*′ to gain token *Tok*. If query attribute set Attr* does not satisfy access policy S*, then challenger C adds *WD*′ to list *L*_*W*_ and sends *Tok* to adversary A.

**Challenge:** The adversary A randomly selects two keyword sets $WD_{0}^{\prime }$ and $WD_{1}^{\prime }$ that are not in list *L*_*W*_. The challenger C throws a fair coin to choose *ξ*∈{0,1}, runs **Keyword-Encrypt** algorithm to gain the index of keyword set $WD_{\xi }^{\prime }$ and transmits it to adversary A.

**Phase 2:** The adversary A repeats queries in Phase 1, but keyword set $WD_{0}^{\prime }$ and $WD_{1}^{\prime }$ can no longer be queried.

**Guess.** Finally, the adversary A gives a guess *ξ*′ of *ξ*. If *ξ*′ = *ξ*, A wins game.

The advantage of adversary A can be defined as

$$ADV\left(A\right)=|\mathrm{Pr}\left[\xi^{\prime }=\xi \right]-\frac{1}{2}|$$

**Definition 3:** If for all polynomial-time adversary who winning game with a negligible advantage, our scheme is called selectively secure against chosen-keyword attacks.

#### 3.2.2 Token privacy game

To ensure the privacy of keyword, an adversary should not infer keyword information from token. In other words, if there is no polynomial-time adversary who can obtain keyword from token, the token privacy security can be guaranteed. The game is set up as follows:

**Setup:** The challenger C runs **Setup** algorithm, generates public key *PK* and master secret key *MSK*, while keeping *MSK* secret.

**Phase 1:** Similar to selectively secure game of chosen-keyword attacks, the challenger C initializes an empty key query list *L*_*K*_, the adversary issues the following polynomial *q*_*t*_ times adaptive queries:

**Outsourced secret key query:** The adversary *A* selects a query attribute set *Attr* for challenger C. The challenger C outputs outsourced secret key *SK*_1_ by running **In-KeyGen** algorithm and **Out-KeyGen** algorithm, then returns *SK*_1_ to adversary A, and the attribute set *Attr* is added to list *L*_*K*_.

**Token query:** The challenger C runs **TokenGen** algorithm based on public key *PK*, outsourced secret key *SK*_1_, and a query keyword set *WD*′ given by adversary, then the challenger C sends token to adversary A.

**Challenge:** The adversary A submits an access policy S* with the restriction that the attribute set *Attr* in list *L*_*K*_ does not satisfy access policy S*. Afterwards, the challenger C randomly chooses keyword set WD*, encrypts it with S* to obtain the index, and then selects an attribute set Attr* such that Attr* satisfies S*. The challenger C executes **In-KeyGen** algorithm, **Out-KeyGen** algorithm and **TokenGen** algorithm to gain the token of keyword set WD*, and transmits token to adversary A.

**Phase 2:** Similar to Phase 1, but keyword set WD* can no longer be inquired.

**Guess:** The adversary A gives a keyword set *WD*″ and forwards it to the challenger C. The challenger C runs **Keyword-Encrypt** algorithm to get the index of keyword set *WD*″, and makes a matching between the token of WD* and the index of *WD*″. If the result returned by **Search** algorithm is 1, then the adversary A wins game.

The advantage of adversary A can be defined at most

$$ADV\left(A\right)=\frac{1}{|\Pi |-q_{t}}+\sigma$$

Because the adversary A wants to gain keyword information from the index, it is necessary to analyze the structure of index. In other words, the adversary A gets information from $H\left(w_{t}^{\prime }\right)$ at most. Due to the unidirectional and collision-resistant properties of the hash function, we assume that the adversary A obtains the advantage of *w*′ from $H\left(w_{t}^{\prime }\right)$ is *σ*, where *σ* is a negligible probability under the security parameter *l*. Π is a keyword space for the selection of keyword set, |Π| represents the size of keyword space and is large enough in practical applications. *q*_*t*_ denotes the number of inquiries about outsourcing private key and token in Phase 1, and *q*_*t*_ is finite size.

The advantage $ADV\left(A\right)=\frac{1}{|\Pi |-q_{t}}+\sigma$ consists of two parts. |Π|−*q*_*t*_ is the size of the remaining keyword set in the keyword space after the phase 1 inquiry. The probability that the adversary A guesses the encrypted keyword from the remaining keyword set is $\frac{1}{\left|\Pi \right|-q_{t}}$. *σ* is the probability that the adversary gets the keyword information from $H\left(w_{t}^{\prime }\right)$. The advantage of adversary A is $\frac{1}{\left|\Pi \right|-q_{t}}+\sigma$ by summing two probabilities. Therefore, the advantage of adversary A can be defined at most $ADV\left(A\right)=\frac{1}{|\Pi |-q_{t}}+\sigma$ which is a negligible advantage.

**Definition 4:** Our proposal is token privacy secure if all polynomial-time adversary have at most a negligible advantage in above game.

#### 3.2.3 Selectively secure game for chosen-plaintext attacks

The section contains two indistinguishable games. Since the process of two games is similar, we only show the security proof of one of the games, the other game is described in detail in the specific security proof phase. The game is as follows:

**Initialization:** The adversary A commits two challenge access policies $S_{0}^{*}$ and $S_{1}^{*}$.

**Setup:** The simulator B runs **Setup** algorithm, produces public key *PK* and master secret key *MSK*, while keeping *MSK* secret and sending *PK* to adversary A.

**Phase 1:** The adversary A chooses a query attribute set *Attr* to simulator B.

Secret key query: If attribute set *Attr* does not satisfy access policies $S_{0}^{*}$ and $S_{1}^{*}$, that is $\gamma \left(Attr,S_{0}^{*}\right)=0\Lambda \gamma \left(Attr,S_{1}^{*}\right)=0$, then simulator B runs **KeyGen** algorithm to obtain local secret key *SK*_2_, and sends to adversary A.

**Challenge:** For access policies $S_{0}^{*}$ and $S_{1}^{*}$, the adversary A submits two equal-length encryption keys *ck*_1_ and *ck*_2_ that are used to encrypt file. Hereafter, the simulator B randomly throws a fair coin to select *b*∈{0,1} and runs **Encryption key-Encrypt** algorithm to gain the ciphertext *CT*′ of encryption key *ck*_*b*_, then transmits *CT*′ to adversary A.

**Phase 2:** Similar to Phase 1, the adversary A continues to query. Nevertheless, the restriction is $\gamma \left(Attr,S_{0}^{*}\right)=0\Lambda \gamma \left(Attr,S_{1}^{*}\right)=0$.

**Guess.** Finally, the adversary A makes a guess *b*′ of *b*. If *b*′ = *b*, A wins game.

The advantage of adversary A to win the game can be defined as

$$ADV\left(A\right)=\left|\mathrm{Pr}\left[b^{\prime }=b\right]-\frac{1}{2}\right|$$

**Definition 5:** Our scheme is selectively secure against chosen-plaintext attacks if for all polynomial-time adversary who could win the game with a negligible advantage.

## Concrete construction

In this section, we take into consideration that most of CP-ABE schemes only support a few functions, which has its limitations for practical application. This motivates us to construct a scheme that supports attribute revocation and multi-keyword search. Meanwhile, our scheme is almost the same as some schemes in terms of calculation amount and calculation time, and even smaller and faster. The following is the specific structure of scheme:

**Setup**(*U*,*l*)→*PK*,*MSK*:This algorithm takes universe set of attributes *U* = {*attr*_1_,*attr*_2_,⋯,*attr*_*n*_} and security parameter *l* as input, it selects a bilinear map $e:G\times G\to G_{T}$, where $G,G_{T}$ are two multiplicative cyclic groups with prime order *p*, *g* is a generator of group G. Let $H:{\left\{0,1\right\}}^{*}\to {\mathbb{Z}}_{p}$ is a one-way hash function. The algorithm chooses *a*,*b*,*c*,*α*,{*r*_1_,*r*_2_,⋯,*r*_2*n*_} from ${\mathbb{Z}}_{p}$ and, {*x*_1_,*x*_2_,⋯,*x*_2*n*_} from G at random, then let *Y* = *e*(*g*,*g*)^*α*^. For each attribute *attr*_*j*_∈*U*(*j*∈[1,*n*]), it picks $vk_{j}\in {\mathbb{Z}}_{p}$ as initial version number, then sets $PK_{j,1}=g^{vk_{j}}$. Since the attribute *attr*_*j*_ has two different values *v*_*j*_ and ^¬^*v*_*j*_, let {*r*_1_,⋯,*r*_*n*_} and {*x*_1_,⋯,*x*_*n*_} denote corresponding parameters when *attr*_*j*_ is equal to *v*_*j*_, {*r*_*n*+1_,⋯,*r*_2*n*_} and {*x*_*n*+1_,⋯,*x*_2*n*_} denote corresponding parameters when *attr*_*j*_ is equal to ^¬^*v*_*j*_. Thus, for all *j* = 1,⋯,*n*, when *attr*_*j*_ is equal to *v*_*j*_, we have $PK_{j,2}=PK_{j,2}^{\prime }=u_{j}=g^{-vk_{j}r_{j}}$, when *attr*_*j*_ is equal to ^¬^*v*_*j*_, we have $PK_{j,2}=PK_{j,2}^{\prime \prime }=u_{j+n}=g^{-vk_{j}r_{j+n}}$. Similarly, the algorithm computes $PK_{j,3}=PK_{j,3}^{\prime }=y_{j}=e{\left(x_{j},g\right)}^{vk_{j}}$ if *attr*_*j*_ is equal to *v*_*j*_, it computes $PK_{j,3}=PK_{j,3}^{\prime \prime }=y_{j+n}=e{\left(x_{j+n},g\right)}^{vk_{j}}$ if *attr*_*j*_ is equal to ^¬^*v*_*j*_. Afterwards, the algorithm randomly selects $\beta \in {\mathbb{Z}}_{p}$, sets $PK_{j,4}=X_{j}=g^{\frac{\beta }{vk_{j}}}$. The public attribute key *PK*_*j*_ is:

$$PK_{j,1}=g^{vk_{j}}$$

$$PK_{j,2}^{\prime }=u_{j}=g^{-vk_{j}r_{j}}$$

$$PK_{j,2}^{\prime \prime }=u_{j+n}=g^{-vk_{j}r_{j+n}}$$

$$PK_{j,3}^{\prime }=y_{j}=e{\left(x_{j},g\right)}^{vk_{j}}$$

$$PK_{j,3}^{\prime \prime }=y_{j+n}=e{\left(x_{j+n},g\right)}^{vk_{j}}$$

$$PK_{j,4}=X_{j}=g^{\frac{\beta }{vk_{j}}}$$

Where *j*∈[1,*n*], $vk_{j}\in {\mathbb{Z}}_{p}$ denotes initial version number of attribute *attr*_*j*_. For simplicity, let

$$PK_{j}={\left\{PK_{j,1},PK_{j,4},\left(PK_{j,2}^{\prime },PK_{j,3}^{\prime }\right)=\left(u_{j},y_{j}\right),\left(PK_{j,2}^{\prime \prime },PK_{j,3}^{\prime \prime }\right)=\left(u_{j+n},y_{j+n}\right)\right\}}_{j\in \left[1,n\right]}$$

Subsequently, the algorithm generates public key *PK* and master secret key *MSK*:

$$PK=\left(e,G,G_{T},g,g^{a},g^{b},g^{c},Y,H,\left\{PK_{j}|attr_{j}\in U\right\}\right)$$

$$MSK=\left(a,b,c,\alpha ,\beta ,{\left\{\left(r_{i},x_{i}\right)\right\}}_{i\in \left[1,2n\right]},\left\{MSK_{j}=vk_{j}|attr_{j}\in U\right\}\right)$$

While keeping *MSK* secret.

**Encrypt**: This algorithm defines an access policy is $S={\tilde{v}}_{1}{\tilde{v}}_{2}\cdots {\tilde{v}}_{n}$, denotes as

$$u_{j}^{\prime }=\left\{ \begin{array}{l} u_{j},\;{\tilde{v}}_{j}=v_{j}\\ u_{j+n},\;{\tilde{v}}_{j}={}^{\neg }v_{j} \end{array}\right.\;j\in \left[1,n\right]$$

The encryption algorithm includes two steps: Step one is to encrypt keyword set, that is to generate an index of keyword set, step two is to encrypt encryption key.

(i) **Keyword-Encrypt**(*PK*,*S*,*WD*)→*Index*
  This algorithm makes public key *PK*, access policy *S* and a keyword set *WD* = {*w*_1_,*w*_2_,⋯*w*_*r*_} extracted from file *M* as input, where *r* is the size of *WD*, namely |*WD*| = *r*. It randomly picks $t_{1},t_{2}\in {\mathbb{Z}}_{p}$, computes $\tilde{u}=g^{t_{2}}\prod_{j=1}^{n}u_{j}^{\prime }$, then sets $W^{\prime }=g^{ct_{1}}$ and $W_{t}=g^{a\left(t_{1}+t_{2}\right)}g^{bH\left(w_{t}\right)t_{1}}$, where *w*_*t*_∈*WD*, *t*∈[1,*r*]. Hereafter, the algorithm outputs the index of keyword set as
  $$Index=\left(\tilde{u},W^{\prime },{\left\{W_{t}\right\}}_{t\in \left[1,r\right]}\right)$$
(ii) **Encryption key-Encrypt**(*PK*,*S*,*ck*)→*CT*′

Before uploading a file *M*, the algorithm encrypts the file as follows:

① It selects an encryption key *ck* from key space, and symmetrically encrypts the file *M* with encryption key *ck* to obtain *E*_*ck*_(*M*).
② It sets an access structure $S={\tilde{v}}_{1}{\tilde{v}}_{2}\cdots {\tilde{v}}_{n}$, encrypts *ck* and outputs the ciphertext of encryption key *ck* through the following steps.

This algorithm makes public key *PK*, access policy *S* and encryption key *ck* as input. It chooses $s\in {\mathbb{Z}}_{p}$ at random, computes *C* = *ck*⋅*Y*^*s*^ and *C*_1_ = *g*^*s*^, then picks up random value $s_{j}\in {\mathbb{Z}}_{p}$ such that $s=\sum_{j=1}^{n}s_{j}$, it computes $C_{j,1}=X_{j}^{s_{j}}$ and $C_{j,2}=u_{j}^{\prime }{}^{s_{j}}$. Consequently, the ciphertext of encryption key *ck* as follows:

$$CT^{\prime }=\left(C,C_{1},{\left\{\left(C_{j,1},C_{j,2}\right)\right\}}_{j\in \left[1,n\right]}\right)$$

Finally, the algorithm outputs the overall ciphertext *Cph* = (*Index*,*E*_*ck*_(*M*),*CT*′).

**In-KeyGen**(*PK*,*MSK*,*Attr*)→*OK*: This algorithm makes public key *PK*, master secret key *MSK* and an attribute set *Attr* as input. It sets *v* = *g*^*ac*^, computes *σ*_*j*_(*j*∈[1,*n*]) according to attribute set $Attr={\hat{v}}_{1}{\hat{v}}_{2}\cdots {\hat{v}}_{n}$. Let

$$\sigma_{j}=\left\{ \begin{array}{l} {\left(x_{j}v^{r_{j}}\right)}^{vk_{j}},\;{\hat{v}}_{j}=v_{j}\\ {\left(x_{j+n}v^{r_{j+n}}\right)}^{vk_{j}},\;{\hat{v}}_{j}={}^{\neg }v_{j} \end{array}\right.\;j\in \left[1,n\right]$$

At last, this algorithm outputs the intermediate key as follows:

$$OK=\left(Attr,v,{\left\{\sigma_{j}\right\}}_{j\in \left[1,n\right]}\right)$$

**Out-KeyGen**(*PK*,*OK*)→*SK*_1_: This algorithm makes public key *PK* and intermediate key *OK* as input. It first computes $\tilde{\sigma }=\prod_{j=1}^{n}\sigma_{j}$, then computes $\tilde{y}=\prod_{j=1}^{n}y_{j}^{\prime }$ based on attribute set $Attr={\hat{v}}_{1}{\hat{v}}_{2}\cdots {\hat{v}}_{n}$, where

$$y_{j}^{\prime }=\left\{ \begin{array}{l} y_{j},\;{\hat{v}}_{j}=v_{j}\\ y_{j+n},\;{\hat{v}}_{j}={}^{\neg }v_{j} \end{array}\right.\;j\in \left[1,n\right]$$

Afterwards, the algorithm outputs the outsourced secret key as

$$SK_{1}=\left(v,\tilde{\sigma },\tilde{y}\right)$$

**KeyGen**(*PK*,*MSK*,*Attr*,*SK*_1_)→*SK*: This algorithm makes public key *PK*, master secret key *MSK*, attribute set *Attr* and outsourced secret key *SK*_1_ as input. It randomly picks $u,\lambda_{j}\in {\mathbb{Z}}_{p}$, where *j* ∈ [1,*n*], the algorithm lets *D*_1_ = *g*^*α*+*βu*^ and $D_{j,1}=X_{j}^{\lambda_{j}}$, it computes $D_{j,2}=g^{vk_{j}\left(u-r_{j}\lambda_{j}\right)}$ when ${\hat{v}}_{j}=v_{j}$, otherwise computes $D_{j,2}=g^{vk_{j}\left(u-r_{j+n}\lambda_{j}\right)}$, then the local secret key as

$$SK_{2}=\left(D_{1},{\left\{\left(D_{j,1},D_{j,2}\right)\right\}}_{j\in \left[1,n\right]}\right)$$

Finally, the algorithm outputs data user's secret key *SK* = (*SK*_1_,*SK*_2_).

**TokenGen**(*PK*,*SK*_1_,*WD*′)→*Tok*: This algorithm makes public key *PK*, outsourced secret key *SK*_1_ and a keyword set $WD^{\prime }=\left\{w_{1}^{\prime },w_{2}^{\prime },\cdots ,w_{d}^{\prime }\right\}$ of interest as input, where the size of *WD*′ is *d*, namely |*WD*′| = *d*. It chooses random value $z\in {\mathbb{Z}}_{p}$, computes $Tok_{1}=\prod_{t=1}^{d}{\left(g^{a}g^{bH\left(w_{t}^{\prime }\right)}\right)}^{z}$ and *Tok*_2_ = *g*^*cz*^. Therefore, the algorithm generates a token of keyword set for data user as follows:

$$Tok=\left(v^{z},{\tilde{\sigma }}^{z},{\tilde{y}}^{z},Tok_{1},Tok_{2}\right)$$

**Search**(*Index*,*Tok*)→{0,1}: Taking as inputs the index relevant to access policy *S* and the token relevant to attribute set *Attr*, this algorithm is executed by cloud sever to test whether there is a matching between the index and the token. In other words, the cloud server determines whether the following equation holds:

$$\frac{e\left(\prod_{l=1}^{d}W_{t_{l}},Tok_{2}\right)}{e\left(W^{\prime },Tok_{1}\right)}\overset{?}{=}\frac{e\left(\tilde{u},v^{z}\right)e\left({\tilde{\sigma }}^{z},g\right)}{{\tilde{y}}^{z}}$$ (1)

In above equation, it involves a matching of the index and the token. In the index generation phase, the data owner encrypts *r* keywords to obtain {*W*_*t*_}_*t*∈[1,*r*]_. In the token generation phase, the data user generates a token for *d* keywords which he is interested in, and computes *Tok*_1_, particularly *d* ≤ *r*. In order to make the Eq (1) can be calculated, the cloud server is to arbitrarily select *d* components from ${\left\{W_{t_{l}}\right\}}_{l\in \left[1,r\right]}$ and execute multiplication operations. In accordance with the theory of probability and mathematical statistics, the total number of such choices is $C_{r}^{d}=\frac{r\left(r-1\right)\left(r-2\right)\cdots \left(r-d+1\right)}{d!}$. Hereafter, the cloud server matches the multiplication of $W_{t_{l}}\left(l\in \left[1,d\right]\right)$ with *Tok*_1_. In the matching of $C_{r}^{d}$ times, as long as there is one successful match, it demonstrates that the above equation holds and the search succeeds.

If and only if the Eq (1) holds, the cloud server returns 1 and transmits overall ciphertext *Cph* = (*Index*,*E*_*ck*_(*M*),*CT*′) to data user, otherwise returns 0.

**Decrypt**(*SK*_2_,*CT*′)→*ck*: This algorithm makes data user's local secret key *SK*_2_ and ciphertext *CT*′ of encryption key as input. If data user's attribute set *Attr* satisfies access policy *S* embedded in the ciphertext, the algorithm decrypts *CT*′ to obtain encryption key *ck* as follows:

$$ck=\frac{C\prod_{j=1}^{n}e\left(C_{j,1},D_{j,2}\right)}{e\left(C_{1},D_{1}\right)\prod_{j=1}^{n}e\left(C_{j,2},D_{j,1}\right)}$$ (2)

Finally, a symmetric decryption algorithm is used to decrypt *E*_*ck*_(*M*) with the encryption key *ck* to gain the file *M*.

**Correctness**: Only when the two conditions *γ*(*Attr*,*S*) = 1 and *WD*′ ⊆ *WD* are both satisfied, the search can succeed. The following is the verification process of Eq (1):

$$\begin{array}{l} \frac{e\left(\prod_{l=1}^{d}W_{t_{l}},Tok_{2}\right)}{e\left(W^{\prime },Tok_{1}\right)}=\frac{e\left(\prod_{t=1}^{d}g^{a\left(t_{1}+t_{2}\right)}g^{bH\left(w_{t}\right)t_{1}},g^{cz}\right)}{e\left(g^{ct_{1}},\prod_{t=1}^{d}g^{az}g^{bzH\left(w_{t}^{\prime }\right)}\right)}\\ \;=\frac{e{\left(g,g\right)}^{at_{1}cz}e{\left(g,g\right)}^{at_{2}cz}e{\left(g,g\right)}^{bt_{1}cz\sum_{t=1}^{d}H\left(w_{t}\right)}}{e{\left(g,g\right)}^{ct_{1}az}e{\left(g,g\right)}^{ct_{1}bz\sum_{t=1}^{d}H\left(w_{t}^{\prime }\right)}}\\ \;=e{\left(g,g\right)}^{t_{2}acz} \end{array}$$

$$\begin{array}{l} \frac{e\left(\tilde{u},v^{z}\right)e\left({\tilde{\sigma }}^{z},g\right)}{{\tilde{y}}^{z}}=\frac{e\left(g^{t_{2}}\prod_{j=1}^{n}u_{j}^{\prime },g^{acz}\right)e\left(\prod_{j=1}^{n}\sigma_{j}^{z},g\right)}{\prod_{j=1}^{n}{y^{\prime }}_{j}^{z}}\\ \;=\frac{e\left(g^{t_{2}}\prod_{j=1}^{n}g^{-vk_{j}r_{i}},g^{acz}\right)e\left(\prod_{j=1}^{n}x_{i}^{vk_{j}z}g^{vk_{j}acr_{i}z},g\right)}{\prod_{j=1}^{n}e{\left(x_{i},g\right)}^{vk_{j}z}}\\ \;=\frac{e{\left(g,g\right)}^{t_{2}acz}\prod_{j=1}^{n}e{\left(g,g\right)}^{-vk_{j}aczr_{i}}\prod_{j=1}^{n}e{\left(x_{i},g\right)}^{vk_{j}z}\prod_{j=1}^{n}e{\left(g,g\right)}^{vk_{j}acr_{i}z}}{\prod_{j=1}^{n}e{\left(x_{i},g\right)}^{vk_{j}z}}\\ \;=e{\left(g,g\right)}^{t_{2}acz} \end{array}$$

Where *i*∈[1,2*n*].

Only when the attribute set of data user satisfies access structure, that is *γ*(*Attr*,*S*) = 1, encryption key *ck* is able to be computed, and the correctness of Eq (2) is verified as follows:

$$\begin{array}{l} \frac{C\prod_{j=1}^{n}e\left(C_{j,1},D_{j,2}\right)}{e\left(C_{1},D_{1}\right)\prod_{j=1}^{n}e\left(C_{j,2},D_{j,1}\right)}=\frac{ck\cdot e{\left(g,g\right)}^{\alpha s}\prod_{j=1}^{n}e\left(X_{j}^{s_{j}},g^{vk_{j}\left(u-r_{i}\lambda_{j}\right)}\right)}{e\left(g^{s},g^{\alpha +\beta u}\right)\prod_{j=1}^{n}e\left(u_{j}^{\prime }{}^{s_{j}},X_{j}^{\lambda_{j}}\right)}\\ \;=\frac{ck\cdot e{\left(g,g\right)}^{\alpha s}\prod_{j=1}^{n}e\left(g^{\frac{\beta s_{j}}{vk_{j}}},g^{vk_{j}\left(u-r_{i}\lambda_{j}\right)}\right)}{e\left(g^{s},g^{\alpha +\beta u}\right)\prod_{j=1}^{n}e\left(g^{-vk_{j}r_{i}s_{j}},g^{\frac{\beta \lambda_{j}}{vk_{j}}}\right)}\\ \;=\frac{ck\cdot e{\left(g,g\right)}^{\alpha s}\prod_{j=1}^{n}e{\left(g,g\right)}^{\beta s_{j}u}\prod_{j=1}^{n}e{\left(g,g\right)}^{-\beta s_{j}r_{i}\lambda_{j}}}{e{\left(g,g\right)}^{s\alpha }e{\left(g,g\right)}^{s\beta u}\prod_{j=1}^{n}e{\left(g,g\right)}^{-r_{i}s_{j}\beta \lambda_{j}}}\\ \;=\frac{ck\cdot e{\left(g,g\right)}^{\beta u\sum_{j=1}^{n}s_{j}}}{e{\left(g,g\right)}^{s\beta u}}\\ \;=ck \end{array}$$

Where *i*∈[1,2*n*].

**Attribute revocation**: For the sake of achieving attribute revocation, the revocation phase is divided into three steps: Attribute authority takes charge of generating update key, non-revoked data user's secret key update, and cloud server is in charge of updating ciphertext. We assume that the *j'*-th attribute *attr*_*j*′_ of data user will be revoked, where *j'* may be any one of 1,⋯,*n*.

i) The attribute authority takes charge of generating update key

When an attribute needs to be revoked, the attribute authority AA inputs the current version number *vk*_*j*′_ of revoked attribute *attr*_*j*_ and chooses a new version number $vk_{j^{\prime }}^{*}$, where $vk_{j^{\prime }}^{*}\in {\mathbb{Z}}_{p}\left(vk_{j^{\prime }}^{*}\neq vk_{j^{\prime }}^{}\right)$, then update key generation algorithm calculates the update key as follows:

$$UK_{j^{\prime }}=\left(UK_{j^{\prime },1}=\frac{vk_{j^{\prime }}^{*}}{vk_{j^{\prime }}},UK_{j^{\prime },2}=\frac{vk_{j^{\prime }}}{vk_{j^{\prime }}^{*}}\right)$$

Afterwards, the attribute authority AA sends *UK*_*j*′_ to key generation server KGS and cloud server CS, and updates partial public attribute key that is related to revoked attribute *attr*_*j*′_ at the same time:

$$PK_{j^{\prime },1}^{*}={\left(PK_{j^{\prime },1}^{}\right)}^{UK_{j^{\prime },1}}=g^{vk_{j^{\prime }}^{*}}$$

$$PK_{j^{\prime },2}^{\prime *}={\left(PK_{j^{\prime },2}^{\prime }\right)}^{UK_{j^{\prime },1}}={\left(u_{j^{\prime }}\right)}^{UK_{j^{\prime },1}}=g^{-vk_{j^{\prime }}^{*}r_{j^{\prime }}}$$

$$PK_{j^{\prime },2}^{\prime \prime *}={\left(PK_{j^{\prime },2}^{\prime \prime }\right)}^{UK_{j^{\prime },1}}={\left(u_{j^{\prime }+n}\right)}^{UK_{j^{\prime },1}}=g^{-vk_{j^{\prime }}^{*}r_{j^{\prime }+n}}$$

$$PK_{j^{\prime },3}^{\prime *}={\left(PK_{j^{\prime },3}^{\prime }\right)}^{UK_{j^{\prime },1}}={\left(y_{j^{\prime }}\right)}^{UK_{j^{\prime },1}}=e{\left(x_{j^{\prime }},g\right)}^{vk_{j^{\prime }}^{*}}$$

$$PK_{j^{\prime },3}^{\prime \prime *}={\left(PK_{j^{\prime },3}^{\prime \prime }\right)}^{UK_{j^{\prime },1}}={\left(y_{j^{\prime }+n}\right)}^{UK_{j^{\prime },1}}=e{\left(x_{j{}^{\prime }+n},g\right)}^{vk_{j^{\prime }}^{*}}$$

$$PK_{j^{\prime },4}^{*}={\left(PK_{j^{\prime },4}\right)}^{UK_{j^{\prime },2}}={\left(X_{j^{\prime }}\right)}^{UK_{j^{\prime },2}}=g^{\frac{\beta }{vk_{j^{\prime }}^{*}}}$$

Consequently, the public attribute key associated with revoked attribute *attr*_*j*′_ as

$$PK_{j^{\prime }}^{*}=\left\{PK_{j^{\prime },1}^{*},PK_{j^{\prime },4}^{*},\left(P{K^{\prime }}_{j^{\prime },2}^{*},P{K^{\prime }}_{j^{\prime },3}^{*}\right),\left(P{K^{\prime \prime }}_{j^{\prime },2}^{*},P{K^{\prime \prime }}_{j^{\prime },3}^{*}\right)\right\}$$

At last, this algorithm generates updated public key PK* and updated master secret key MSK*:

$$PK^{*}=\left(e,G,G_{T},g,g^{a},g^{b},g^{c},Y,H,PK_{j^{\prime }}^{*},\left\{PK_{j}|attr_{j}\in U,j\neq j^{\prime }\right\}\right)$$

$$MSK^{*}=\left(a,b,c,\alpha ,\beta ,{\left\{\left(r_{i},x_{i}\right)\right\}}_{i\in \left[1,2n\right]},MSK_{j^{\prime }}^{*}=vk_{j^{\prime }}^{*},\left\{MSK_{j}=vk_{j}|attr_{j}\in U,j\neq j^{\prime }\right\}\right)$$

ii) The non-revoked data user's secret key update

Firstly, attribute authority AA updates the partial intermediate key *OK* that is related to revoked attribute *attr*_*j*′_, this algorithm computes

$$\sigma_{j^{\prime }}^{*}={\left(\sigma_{j^{\prime }}\right)}^{UK_{j^{\prime },1}}=\left\{ \begin{array}{l} {\left(x_{j^{\prime }}v^{r_{j^{\prime }}}\right)}^{vk_{j^{\prime }}^{*}},\;{\hat{v}}_{j^{\prime }}=v_{j^{\prime }}\\ {\left(x_{j^{\prime }+n}v^{r_{j^{\prime }}+n}\right)}^{vk_{j^{\prime }}^{*}},\;{\hat{v}}_{j^{\prime }}={}^{\neg }v_{j^{\prime }} \end{array}\right.$$

And transmits $\sigma_{j^{\prime }}^{*}$ to key generation server KGS.

Secondly, AA updates the partial local secret key *SK*_2_ related to revoked attribute *attr*_*j*′_ according to *UK*_*j*′_ as follows:

$$\begin{array}{l} SK_{2}^{*}=(D_{1},D_{j^{\prime },1}^{*}={\left(D_{j^{\prime },1}\right)}^{UK_{j^{\prime },2}}=g^{\frac{\beta \lambda_{j^{\prime }}}{vk_{j^{\prime }}^{*}}},D_{j^{\prime },2}^{*},\\ \;\forall attr_{j}\in Attr\backslash \left\{attr_{j^{\prime }}\right\}:D_{j,1},D_{j,2}) \end{array}$$

where

$$D_{j^{\prime },2}^{*}={\left(D_{j^{\prime },2}\right)}^{UK_{j^{\prime },1}}=\left\{ \begin{array}{l} g^{vk_{j^{\prime }}^{*}\left(u-r_{j^{\prime }}\lambda_{j^{\prime }}\right)},\;{\hat{v}}_{j^{\prime }}=v_{j^{\prime }}\\ g^{vk_{j^{\prime }}^{*}\left(u-r_{j^{\prime }+n}\lambda_{j^{\prime }}\right)},\;{\hat{v}}_{j^{\prime }}={}^{\neg }v_{j^{\prime }} \end{array}\right.$$

Note that only partial local secret key that is related to revoked attribute *attr*_*j*′_ will be updated, others remain unchanged.

Thirdly, KGS updates the partial outsourced secret key that is related to revoked attribute *attr*_*j*′_, the algorithm computes:

$${\tilde{\sigma }}^{*}=\prod_{\begin{array}{l} j=1\\ j\neq j^{\prime } \end{array}}^{n}\sigma_{j}\cdot \sigma_{j^{\prime }}^{*}$$

$$y_{j^{\prime }}^{\prime *}={\left(y_{j^{\prime }}^{\prime }\right)}^{UK_{j^{\prime },1}}=\left\{ \begin{array}{l} {\left(y_{j^{\prime }}\right)}^{UK_{j^{\prime },1}}=e{\left(x_{j^{\prime }},g\right)}^{vk_{j^{\prime }}^{*}},{\hat{v}}_{j^{\prime }}=v_{j^{\prime }}\\ {\left(y_{j^{\prime }+n}\right)}^{UK_{j^{\prime },1}}=e{\left(x_{j^{\prime }+n},g\right)}^{vk_{j^{\prime }}^{*}},{\hat{v}}_{j^{\prime }}={}^{\neg }v_{j^{\prime }} \end{array}\right.$$

$${\tilde{y}}^{*}=\prod_{\begin{array}{l} j=1\\ j\neq j^{\prime } \end{array}}^{n}y_{j}^{\prime }\cdot {y^{\prime }}_{j^{\prime }}^{*}$$

Then, it returns $\left({\tilde{\sigma }}^{*},{\tilde{y}}^{*}\right)$ to AA.

Finally, AA sends the updated secret key $SK^{*}=\left(SK_{1}^{*}=\left(v,{\tilde{\sigma }}^{*},{\tilde{y}}^{*}\right),SK_{2}^{*}\right)$ to non-revoked data user. In the meanwhile, since token contains the component of secret key, then non-revoked data user's token is updated as

$$Tok^{*}=\left(v^{z},{\tilde{\sigma }}^{*z},{\tilde{y}}^{*z},Tok_{1},Tok_{2}\right).$$

iii) The cloud server is in charge of updating ciphertext

① The update of keyword index

Based on updated key *UK*_*j*′_ sent by AA, the cloud server updates partial keyword index that is related to revoked attribute *attr*_*j*′_, this algorithm computes:

$$u_{j^{\prime }}^{\prime *}={\left(u_{j^{\prime }}^{\prime }\right)}^{UK_{j^{\prime },1}}=\left\{ \begin{array}{l} {\left(u_{j^{\prime }}\right)}^{UK_{j^{\prime },1}}=g^{-vk_{j^{\prime }}^{*}r_{j^{\prime }}},\;{\tilde{v}}_{j^{\prime }}=v_{j^{\prime }}\\ {\left(u_{j^{\prime }+n}\right)}^{UK_{j^{\prime },1}}=g^{-vk_{j^{\prime }}^{*}r_{j^{\prime }+n}},\;{\tilde{v}}_{j^{\prime }}={}^{\neg }v_{j^{\prime }} \end{array}\right.$$

$${\tilde{u}}^{*}=g^{t_{2}}\prod_{\begin{array}{l} j=1\\ j\neq j^{\prime } \end{array}}^{n}u_{j}^{\prime }\cdot u_{j^{\prime }}^{\prime *}$$

Hereafter, the updated keyword index is $Index^{*}=\left({\tilde{u}}^{*},W^{\prime },W\right)$.

② The ciphertext update of encryption key

The cloud server CS updates the partial ciphertext of encryption key related to revoked attribute *attr*_*j*′_, the updated ciphertext as follows:

$$\begin{array}{l} C{T^{\prime }}^{**}=(C,C_{1},C_{j^{\prime },1}^{**}={\left(C_{j^{\prime },1}\right)}^{UK_{j^{\prime },2}}=g^{\frac{\beta s_{j^{\prime }}}{vk_{j^{\prime }}^{*}}},C_{j^{\prime },2}^{**},\\ \forall attr_{j}\in Attr\backslash \left\{attr_{j^{\prime }}\right\}:C_{j,1},C_{j,2}) \end{array}$$

where

$$C_{j^{\prime },2}^{**}={\left(C_{j^{\prime },2}\right)}^{UK_{j^{\prime },1}}=\left\{ \begin{array}{l} g^{-vk_{j^{\prime }}^{*}r_{j^{\prime }}s_{j^{\prime }}},\;{\tilde{v}}_{j^{\prime }}=v_{j^{\prime }}\\ g^{-vk_{j^{\prime }}^{*}r_{j^{\prime }+n}s_{j^{\prime }}},\;{\tilde{v}}_{j^{\prime }}={}^{\neg }v_{j^{\prime }} \end{array}\right.$$

Note that only partial ciphertext that is related to revoked attribute *attr*_*j*′_ will be updated, others remain unchanged.

Finally, the updated overall ciphertext is Cph* = (Index*,*E*_*ck*_(*M*),*CT*^′**^).

## Security proof

### 5.1 Selectively secure game of chosen-keyword attacks

**Theorem 1.** If there is an adversary A who is able to win selectively secure game of chosen-keyword attacks with advantage *ε*, then advantage of a challenger C to break the DL assumption can be defined as $\frac{\varepsilon }{2}$.

Poof: Supposing that a tuple $\left(g,f,h,f^{r_{1}},g^{r_{2}},R\right)$ which is an instance of the DL problem, where g,f,h,R∈G, $r_{1},r_{2}\in {\mathbb{Z}}_{p}^{*}$, is given to a challenger C, the challenger C performs the following selectively secure game of chosen-keyword attacks with an adversary A, we will proof that if the adversary A who can break our scheme with advantage *ε*, then the challenger C can solve the DL problem with advantage $\frac{\varepsilon }{2}$.

**Setup.** The challenger C runs **Setup** algorithm, selects a bilinear map $e:G\times G\to G_{T}$, where $G,G_{T}$ are two multiplicative cyclic groups with prime order *p*, *g* is a generator of group G. Let $H:{\left\{0,1\right\}}^{*}\to {\mathbb{Z}}_{p}$ is a one-way hash function. It randomly picks $\left\{r_{1},r_{2},\cdots ,r_{2n}\right\}\in {\mathbb{Z}}_{p}$, $\left\{x_{1},x_{2},\cdots ,x_{2n}\right\}\in {\mathbb{Z}}_{p}$, and implicitly sets *h* = ^*ga*^,*f* = *g*^*c*^, it chooses $d\in {\mathbb{Z}}_{p}$ at random, lets *f*^*d*^ = *g*^*b*^, where implies that *b* = *cd*. For each attribute *attr*_*j*_∈*U*(*j*∈[1,*n*]), the challenger C chooses $vk_{j}\in {\mathbb{Z}}_{p}$ as initial version number, when *attr*_*j*_ is equal to *v*_*j*_, we have $u_{j}=g^{-vk_{j}r_{j}}$, when *attr*_*j*_ is equal to ^¬^*v*_*j*_, we have $u_{j+n}=g^{-vk_{j}r_{j+n}}$. Similarly, C computes $y_{j}=e{\left(x_{j},g\right)}^{vk_{j}}$ if *attr*_*j*_ is equal to *v*_*j*_, it computes $y_{j+n}=e{\left(x_{j+n},g\right)}^{vk_{j}}$ if *attr*_*j*_ is equal to ^¬^*v*_*j*_. The public key and master secret key are

$$PK=\left(e,G,G_{T},g,h,f^{d},f,H,{\left\{\left(u_{j},y_{j}\right),\left(u_{j+n},y_{j+n}\right)\right\}}_{j\in \left[1,n\right]}\right)$$

$$MSK=\left(a,c,d,{\left\{\left(r_{i},x_{i}\right)\right\}}_{i\in \left[1,2n\right]},{\left\{vk_{j}\right\}}_{j\in \left[1,n\right]}\right)$$

The adversary A submits a challenge access policy $S^{*}={\tilde{v}}_{1}{\tilde{v}}_{2}\cdots {\tilde{v}}_{n}$ to the challenger C.

**Phase 1.** The challenger C initializes an empty keyword set query list *L*_*W*_, then adversary A issues the following polynomial times adaptive queries:

Outsourced secret key query: The adversary A sends a query attribute set $Attr^{*}={\hat{v}}_{1}{\hat{v}}_{2}\cdots {\hat{v}}_{n}$ to challenger C, where ${\hat{v}}_{j}=v_{j}$ or ${\hat{v}}_{j}={}^{\neg }v_{j}$.

① If *γ*(Attr*,S*) = 1, the game outputs termination.
② If *γ*(Attr*,S*) = 0, the challenger C computes *v* = *f*^*k*′^, implicitly sets *k*′ = *a*, then it computes $\tilde{\sigma }=\prod_{j=1}^{n}\sigma_{j}$, $\tilde{y}=\prod_{j=1}^{n}y_{j}^{\prime }$, where
  $$\sigma_{j}=\left\{ \begin{array}{l} {\left(x_{j}v^{r_{j}}\right)}^{vk_{j}},\;{\hat{v}}_{j}=v_{j}\\ {\left(x_{j+n}v^{r_{j+n}}\right)}^{vk_{j}},\;{\hat{v}}_{j}={}^{\neg }v_{j} \end{array}\right.\;j\in \left[1,n\right]$$
  $$y_{j}^{\prime }=\left\{ \begin{array}{l} y_{j},\;{\hat{v}}_{j}=v_{j}\\ y_{j+n},\;{\hat{v}}_{j}={}^{\neg }v_{j} \end{array}\right.\;j\in \left[1,n\right]$$
  Subsequently, the challenger C transmits $SK_{1}=\left(v,\tilde{\sigma },\tilde{y}\right)$ to adversary A.

Token query: The adversary A commits a query keyword set $WD^{\prime }=\left({\bar{w}}_{1}^{\prime },{\bar{w}}_{2}^{\prime },\cdots ,{\bar{w}}_{d}^{\prime }\right)$. The challenger C randomly selects $\eta \in {\mathbb{Z}}_{p}$ and generates the token of keyword set *WD*′, that is

$$Tok=\left(Tok_{1}=\prod_{t=1}^{d}{\left(hf^{dH\left({\bar{w}}_{t}^{\prime }\right)}\right)}^{\eta },Tok_{2}=f^{\eta },v^{\eta },{\tilde{\sigma }}^{\eta },{\tilde{y}}^{\eta }\right)$$

If the query attribute set Attr* does not satisfy access policy S*, then the challenger C adds *WD*′ to list *L*_*W*_ and sends *Tok* to adversary A.

**Challenge.** The adversary A randomly picks two keyword sets $WD_{0}^{\prime }=\left({\bar{w}}_{0,1}^{\prime },{\bar{w}}_{0,2}^{\prime },\cdots {\bar{w}}_{0,r}^{\prime }\right)$ and $WD_{1}^{\prime }=\left({\bar{w}}_{1,1}^{\prime },{\bar{w}}_{1,2}^{\prime },\cdots {\bar{w}}_{1,r}^{\prime }\right)$ that are not in list *L*^*W*^, and forwards to challenger C. The challenger C throws a fair coin to select *ξ*∈{0,1}, runs **keyword-Encrypt** algorithm, and computes

$$\tilde{u}=g^{t_{2}}\prod_{j=1}^{n}u_{j}^{\prime }=g^{r_{2}}\prod_{j=1}^{n}u_{j}^{\prime }$$

$$W^{\prime }=f^{t_{1}}=f^{r_{1}}$$

$$W_{t}=Rf^{dH\left({{\bar{w}}^{\prime }}_{\xi ,\;t}\right)r_{1}},t\in \left[1,r\right]$$

which implies *t*_1_ = *r*_1_,*t*_2_ = *r*_2_, where

$$u_{j}^{\prime }=\left\{ \begin{array}{l} u_{j},\;{\tilde{v}}_{j}=v_{j}\\ u_{j+n},\;{\tilde{v}}_{j}={}^{\neg }v_{j} \end{array}\right.\;j\in \left[1,n\right]$$

As such, the keyword index is $Index=\left(\tilde{u},W^{\prime },{\left\{W_{t}\right\}}_{t\in \left[1,r\right]}\right)$, then the challenger C returns *Index* to adversary A.

**Phase 2.** Except that the keyword set $WD_{0}^{\prime }$ and $WD_{1}^{\prime }$ can no longer be queried, the other queries are similar to Phase 1.

**Guess.** Finally, the adversary A makes a guess *ξ*′ of *ξ*. If *ξ*′ = *ξ*, A wins game. The challenger C considers $R=h^{r_{1}+r_{2}}$ implicitly. Because when $R=h^{r_{1}+r_{2}}$, *Index* is a legitimate index of keyword set $WD_{\xi }^{\prime }$.

Afterwards, we assume that the adversary A who can break the scheme with non-negligible advantage *ε*, the probability is $\mathrm{Pr}\left[\xi^{\prime }=\xi \right]=\frac{1}{2}+\varepsilon$ if $R=h^{r_{1}+r_{2}}$, otherwise $\mathrm{Pr}\left[\xi^{\prime }=\xi \right]=\frac{1}{2}$. The advantage of challenger C in solving DL assumption is defined as

$$\begin{array}{l} ADV\left(C\right)=\left\{\frac{1}{2}\mathrm{Pr}\left[\xi^{\prime }=\xi |R=h^{r_{1}+r_{2}}\right]+\frac{1}{2}\mathrm{Pr}\left[\xi^{\prime }=\xi |R\neq h^{r_{1}+r_{2}}\right]\right\}-\frac{1}{2}\\ \;=\frac{1}{2}\times \left(\frac{1}{2}+\varepsilon \right)+\frac{1}{2}\times \frac{1}{2}-\frac{1}{2}\\ \;=\frac{\varepsilon }{2} \end{array}$$

Therefore, our scheme will achieve selectively secure against chosen-keyword attacks.

### 5.2 Token privacy game

**Theorem 2.** If there is an adversary A who is able to break token privacy game with a non-negligible advantage, a challenger C is capable of obtaining $w_{t}^{\prime }$ from $H\left(w_{t}^{\prime }\right)$ with a non-negligible advantage.

Proof: Here we use token privacy game to prove the theorem.

**Setup.** The challenger C executes **Setup** algorithm, selects a bilinear map $e:G\times G\to G_{T}$, where $G,G_{T}$ are two multiplicative cyclic groups with prime order *p*, *g* is a generator of group G. Let $H:{\left\{0,1\right\}}^{*}\to {\mathbb{Z}}_{p}$ is a one-way hash function. It randomly chooses $a,b,c,{\left\{r_{i}\right\}}_{i\in \left[1,2n\right]}\leftarrow {\mathbb{Z}}_{p}$ and ${\left\{x_{i}\right\}}_{i\in \left[1,2n\right]}\leftarrow G$. For each attribute *attr*_*j*_∈*U*, $vk_{j}\in {\mathbb{Z}}_{p}$ is initial version number, where *j*∈[1,*n*]. When *attr*_*j*_ is equal to *v*_*j*_, we have $u_{j}=g^{-vk_{j}r_{j}}$, when *attr*_*j*_ is equal to ^¬^*v*_*j*_, we have $u_{j+n}=g^{-vk_{j}r_{j+n}}$. Similarly, C computes $y_{j}=e{\left(x_{j},g\right)}^{vk_{j}}$ if *attr*_*j*_ is equal to *v*_*j*_, it computes $y_{j+n}=e{\left(x_{j+n},g\right)}^{vk_{j}}$ if *attr*_*j*_ is equal to ^¬^*v*_*j*_. The challenger C generates public key $PK=\left(e,G,G_{T},g,g^{a},g^{b},g^{c},H,{\left\{\left(u_{j},y_{j}\right),\left(u_{j+n},y_{j+n}\right)\right\}}_{j\in \left[1,n\right]}\right)$, master secret key *MSK* = (*a*,*b*,*c*{(*r*_*i*_,*x*_*i*_)}_*i*∈[2*n*]_,{*vk*_*j*_}_*j*∈[1,*n*]_), while publishing *PK* and keeping *MSK* secret.

**Phase 1.** Similar to selectively secure game of chosen-keyword attacks, the challenger C initializes an empty key query list *L*_*K*_, the adversary issues the following polynomial *q*_*t*_ times adaptive queries:

Outsourced secret key query: The adversary A selects a query attribute set $Attr={\hat{v}}_{1}{\hat{v}}_{2}\cdots {\hat{v}}_{n}$ as input for **In-KeyGen** algorithm, where ${\hat{v}}_{j}=v_{j}$ or ${\hat{v}}_{j}={}^{\neg }v_{j}$, the challenger C computes *v* = *g*^*ac*^ and

$$\sigma_{j}=\left\{ \begin{array}{l} {\left(x_{j}v^{r_{j}}\right)}^{vk_{j}},\;{\hat{v}}_{j}=v_{j}\\ {\left(x_{j+n}v^{r_{j+n}}\right)}^{vk_{j}},\;{\hat{v}}_{j}={}^{\neg }v_{j} \end{array}\right.\;j\in \left[1,n\right]$$

Hereafter, it obtains the intermediate key

$$OK=\left(Attr,v,{\left\{\sigma_{j}\right\}}_{j\in \left[1,n\right]}\right)$$

The challenger C runs **Out-KeyGen** algorithm to output the outsourced secret key $SK_{1}=\left(v,\tilde{\sigma }=\prod_{j=1}^{n}\sigma_{j},\tilde{y}=\prod_{j=1}^{n}y_{j}^{\prime }\right)$, where

$$y_{j}^{\prime }=\left\{ \begin{array}{l} y_{j},\;{\hat{v}}_{j}=v_{j}\\ y_{j+n},\;{\hat{v}}_{j}={}^{\neg }v_{j} \end{array}\right.\;j\in \left[1,n\right]$$

Then, the challenger C transmits *SK*_1_ to adversary A, and adds *Attr* to list *L*_*K*_.

Token query: The challenger C runs **TokenGen** algorithm based on outsourced secret key *SK*_1_ and keyword set $WD^{\prime }=\left(w_{1}^{\prime },w_{2}^{\prime },\cdots ,w_{d}^{\prime }\right)$ submitted by adversary A, then it randomly picks $z^{\prime }\in {\mathbb{Z}}_{p}$ to compute $Tok_{1}=\prod_{t=1}^{d}{\left(g^{a}g^{bH\left(w_{t}^{\prime }\right)}\right)}^{z^{\prime }}$ and *Tok*_2_ = *g*^*cz*′^, sends the token $Tok=\left(v^{z^{\prime }},{\tilde{\sigma }}^{z^{\prime }},{\tilde{y}}^{z^{\prime }},Tok_{1},Tok_{2}\right)$ to adversary A.

**Challenge.** The adversary A submits an access policy S* with the restriction that the attribute set *Attr* in list *L*_*K*_ does not satisfy access policy S*, namely *γ*(*Attr*,S*) = 0. The challenger C randomly selects a keyword set WD*, encrypts WD* with S* to obtain Index*, then it chooses an attribute set Attr* such that Attr* satisfies access policy S*, that is *γ*(Attr*,S*) = 1. Subsequently, the challenger C first executes **In-KeyGen** algorithm and **Out-KeyGen** algorithm to compute $SK_{1}^{*}$, then runs **TokenGen** algorithm to gain Tok*, and sends Tok* to adversary A.

**Phase 2.** Similar to Phase 1, but keyword set WD* can no longer be queried.

**Guess.** The adversary A outputs a keyword set *WD*″ and transmits to challenger C. If *WD*″ = WD*, adversary A wins game. In other words, the challenger C performs **Keyword-Encrypt** algorithm to generate the ciphertext *Index*″of *WD*″, if the result returned by **Search**(*Index*″,Tok*) algorithm is 1, then the adversary A wins game.

Particularly, if adversary A attempts to gain keyword information from the index, it needs to analyze $Tok_{1}=\prod_{t=1}^{d}{\left(g^{a}g^{bH\left(w_{t}^{\prime }\right)}\right)}^{z^{\prime }}$, where *z*′ is randomly selected by challenger C, thus the adversary A gets information from $H\left(w_{t}^{\prime }\right)$ at most. Due to the unidirectional and collision-resistant properties of the hash function, if adversary A obtains the advantage of $w_{t}^{\prime }$ from $H\left(w_{t}^{\prime }\right)$ is negligible *σ*, |Π| represents the size of keyword space, *q*_*t*_ represents the number of inquiries of Phases 1, |Π|−*q*_*t*_ is the size of the remaining keyword set in the keyword space after the phase 1 inquiry. The probability that the adversary A guesses the encrypted keyword from the remaining keyword set is $\frac{1}{\left|\Pi \right|-q_{t}}$. Then, the advantage of adversary A to break token privacy game can be defined at most $ADV\left(A\right)=\frac{1}{\left|\Pi \right|-q_{t}}+\sigma$. In practical applications, because |Π| is large enough and *q*_*t*_ is finite size, then the advantage $ADV\left(A\right)=\frac{1}{\left|\Pi \right|-q_{t}}+\sigma$ is negligible, therefore our proposal can guarantee token privacy security.

### 5.3 Selectively secure game of chosen-plaintext attacks

Our scheme is selective security under the assumption. For selective security, the adversary A should submit two challenge access policies $S_{0}^{*}$ and $S_{1}^{*}$ at the beginning of game, then the scheme is to prove that the adversary A cannot win the game with a non-negligible advantage through the indistinguishability of a series of games. In simple terms, we use $\left(C^{*},C_{1}^{*},{\left\{\left(C_{j,1}^{*},C_{j,2}^{*}\right)\right\}}_{j\in \left[1,n\right]}\right)$ to denote initial challenge ciphertext, then select random element *Z* in $G_{T}$ and {*Z*_*j*,1_}_*j*∈[1,*n*]_ in G. Afterwards, we define a series of games *Game*_0_,⋯,*Game*_*n*+1_, where *Game*_0_ is initial game, *Game*_1_ is to replace C* in *Game*_0_ with *Z*, *Game*_2_,⋯,*Game*_*n*+1_ are to replace $C_{j,1}^{*}$ with *Z*_*j*,1_, for *j*∈[1,*n*]. The game is defined as follows:

*Game*_0_:The challenge ciphertext is $CT_{0}^{\prime *}=\left(C^{*},C_{1}^{*},{\left\{\left(C_{j,1}^{*},C_{j,2}^{*}\right)\right\}}_{j\in \left[1,n\right]}\right)$
*Game*_1_:The challenge ciphertext is $CT_{1}^{\prime *}=\left(Z,C_{1}^{*},{\left\{\left(C_{j,1}^{*},C_{j,2}^{*}\right)\right\}}_{j\in \left[1,n\right]}\right)$
*Game*_2_:The challenge ciphertext is $CT_{2}^{\prime *}=\left(Z,C_{1}^{*},\left(Z_{1,1},C_{1,2}^{*}\right),{\left\{\left(C_{j,1}^{*},C_{j,2}^{*}\right)\right\}}_{j\in \left[2,n\right]}\right)$
……
*Game*_*n*+1_:The challenge ciphertext is $CT_{n+1}^{\prime *}=\left(Z,C_{1}^{*},{\left\{\left(Z_{j,1},C_{j,2}^{*}\right)\right\}}_{j\in \left[1,n\right]}\right)$

When a data user's attribute set *Attr* satisfies both access structures $S_{0}^{*}$ and $S_{1}^{*}$, namely $\gamma \left(Attr,S_{0}^{*}\right)=1\Lambda \gamma \left(Attr,S_{1}^{*}\right)=1$, *Game*_1_ is the same as *Game*_0_. When a data user's attribute set *Attr* does not satisfy access policies $S_{0}^{*}$ and $S_{1}^{*}$, that is $\gamma \left(Attr,S_{0}^{*}\right)=0\Lambda \gamma \left(Attr,S_{1}^{*}\right)=0$, *Game*_1_ is as defined above. Subsequently, we use the DDH assumption to prove that *Game*_0_ and *Game*_1_ are indistinguishable.

**Theorem 3.** If there is an adversary A who is able to distinguish games *Game*_0_ and *Game*_1_ with a non-negligible advantage, a simulator B is capable of breaking DDH assumption with a non-negligible advantage.

Proof: Supposing that a tuple $\left(g,g^{z_{1}},g^{z_{2}},Q\right)$ which is an instance of DDH problem, where g,Q∈G, $z_{1},z_{2}\in {\mathbb{Z}}_{p}^{*}$, is given to a simulator B, the simulator B performs the following operations:

**Initialization.** The adversary A submits two challenge access policies $S_{0}^{*}={\tilde{v}}_{0,1}{\tilde{v}}_{0.2}\cdots {\tilde{v}}_{0,n}$ and $S_{1}^{*}={\tilde{v}}_{1,1}{\tilde{v}}_{1,2}\cdots {\tilde{v}}_{1,n}$ to simulator B, then B throws a fair coin to select *b*∈{0,1}.

**Setup.** The simulator B runs **Setup** algorithm, selects a bilinear map $e:G\times G\to G_{T}$, where $G,G_{T}$ are two multiplicative cyclic groups with prime order *p*, *g* is a generator of group G. It randomly chooses $\alpha ,\beta ,vk_{j}\in {\mathbb{Z}}_{p}$, sets $Y=e{\left(g,g\right)}^{\alpha }=e\left(g,g^{z_{1}}\right)=e{\left(g,g\right)}^{z_{1}}$, implicitly lets *α* = *z*_1_, then it computes $X_{j}=g^{\frac{\beta }{vk_{j}}}$, where $vk_{j}\in {\mathbb{Z}}_{p}$ as initial version number of attribute, for *j*∈[1,*n*]. Afterwards, B picks up random value ${\left\{r_{i}\in {\mathbb{Z}}_{p}\right\}}_{i\in \left[1,2n\right]}$, when ${\hat{v}}_{j}^{}\in S_{b}^{*}$, there are $u_{j}=g^{-vk_{j}r_{j}}\left({\hat{v}}_{j}=v_{j}={\tilde{v}}_{j}\right)$ and $u_{j+n}=g^{-vk_{j}r_{j+n}}\left({\hat{v}}_{j}={}^{\neg }v_{j}={\tilde{v}}_{j}\right)$, when ${\hat{v}}_{j}^{}\notin S_{b}^{*}$, there are ${\bar{u}}_{j}=g^{-z_{1}vk_{j}r_{j}}\left({\hat{v}}_{j}=v_{j}\neq {\tilde{v}}_{j}\right)$ and ${\bar{u}}_{j+n}=g^{-z_{1}vk_{j}r_{j+n}}\left({\hat{v}}_{j}={}^{\neg }v_{j}\neq {\tilde{v}}_{j}\right)$, where *j*∈[1,*n*]. The simulator B publishes public key

$$PK=\left(e,G,G_{T},g,Y,{\left\{\left(X_{j},u_{j},u_{j+n},{\bar{u}}_{j},{\bar{u}}_{j+n}\right)\right\}}_{j\in \left[1,n\right]}\right)$$

and keeps master secret key *MSK* = (*α,β*,{*vk_j_*,}_*j*∈[1,*n*]_,{*r_i_*}_*i*∈[1,2*n*]_) secret.

**Phase 1.** The adversary A commits a query attribute set $Attr={\hat{v}}_{1}{\hat{v}}_{2}\cdots {\hat{v}}_{n}$ to simulator B.

Secret key query: Considering that $\gamma \left(Attr,S_{0}^{*}\right)=0\Lambda \gamma \left(Attr,S_{1}^{*}\right)=0$, thus there is *j*″∈{1,2,⋯,*n*} such that ${\hat{v}}_{j^{\prime \prime }}\notin S_{b}^{*}$. Then, the simulator B picks up $u,\lambda_{j}\in {\mathbb{Z}}_{p}$ at random, *D*_1_ and *D*_*j*,1_ in the local secret key *SK*_2_ are calculated as $D_{1}=g^{\alpha +\beta u}=g^{z_{1}+\beta u}$ and $D_{j,1}=X_{j}^{\lambda_{j}}=g^{\frac{\beta \lambda_{j}}{vk_{j}}}$, where *j*∈[1,*n*]. For *j* = *j*″, B computes

$$D_{j^{\prime \prime },2}=\left\{ \begin{array}{l} {\bar{u}}_{j^{\prime \prime }}^{\lambda_{j^{\prime \prime }}}g^{vk_{j^{\prime \prime }}u}={\left(g^{-z_{1}}\right)}^{vk_{j^{\prime \prime }}r_{j^{\prime \prime }}\lambda_{j^{\prime \prime }}}g^{vk_{j^{\prime \prime }}u},\;{\hat{v}}_{j^{\prime \prime }}=v_{j^{\prime \prime }}\\ {\bar{u}}_{j^{\prime \prime }+n}^{\lambda_{j^{\prime \prime }}}g^{vk_{j^{\prime \prime }}u}={\left(g^{-z_{1}}\right)}^{vk_{j^{\prime \prime }}r_{j^{\prime \prime }+n}\lambda_{j^{\prime \prime }}}g^{vk_{j^{\prime \prime }}u},\;{\hat{v}}_{j^{\prime \prime }}={}^{\neg }v_{j^{\prime \prime }} \end{array}\right.$$

for *j* ≠ *j*″, B computes

$$D_{j,2}=\left\{ \begin{array}{l} u_{j}^{\lambda_{j}}g^{vk_{j}u}=g^{-vk_{j}r_{j}\lambda_{j}}g^{vk_{j}u},\;{\hat{v}}_{j}=v_{j}\\ u_{j+n}^{\lambda_{j}}g^{vk_{j}u}=g^{-vk_{j}r_{j+n}\lambda_{j}}g^{vk_{j}u},\;{\hat{v}}_{j}={}^{\neg }v_{j} \end{array}\right.$$

Afterwards, the simulator B returns *SK*_2_ = (*D*_1_,{(*D*_*j*,1_,*D*_*j*,2_)}_*j*∈[1,*n*]_) to adversary A.

**Challenge.** For access policies $S_{0}^{*}={\tilde{v}}_{0,1}{\tilde{v}}_{0.2}\cdots {\tilde{v}}_{0,n}$ and $S_{1}^{*}={\tilde{v}}_{1,1}{\tilde{v}}_{1,2}\cdots {\tilde{v}}_{1,n}$, the adversary A submits two equal-length encryption keys *ck*_1_ and *ck*_2_. Then, the simulator B executes **Encryption key-Encrypt** algorithm with $S_{b}^{*}$ and *ck*_*b*_, where *b*∈{0,1}. It sets $C_{1}^{*}=g^{s}=g^{z_{2}}$, C* = *ck*_*b*_⋅*Y*^*s*^ = *ck*_*b*_⋅*e*(*g*,*g*)^*αs*^ = *ck*_*b*_⋅*e*(*g*,*Q*), implicitly lets *s* = *z*_2_. For all *j*∈[1,*n*], the simulator B arbitrarily chooses $s_{j}\in {\mathbb{Z}}_{p}$ if *j* ≠ *j*″, it computes $s_{j^{\prime \prime }}=z_{2}-\sum_{j=1,j\neq j^{\prime \prime }}^{n}s_{j}$ if *j* = *j*″.

When *j* ≠ *j*″, $\left[C_{j^{\prime \prime },1}^{*},C_{j^{\prime \prime },2}^{*}\right]$ in the ciphertext are computed as

$$C_{j^{\prime \prime },1}^{*}=X_{j^{\prime \prime }}^{s_{j^{\prime \prime }}}=g^{\frac{\beta }{vk_{j^{\prime \prime }}}\left(z_{2}-\sum_{j=1,j\neq j^{\prime \prime }}^{n}s_{j}\right)}={\left(g^{z_{2}}\right)}^{\frac{\beta }{vk_{j^{\prime \prime }}}}/g^{\frac{\beta }{vk_{j^{\prime \prime }}}\sum_{j=1,j\neq j^{\prime \prime }}^{n}s_{j}}$$

$$C_{j^{\prime \prime },2}^{*}=u_{j^{\prime \prime }}^{\prime }{}^{s_{j^{\prime \prime }}}=\left\{ \begin{array}{l} {\bar{u}}_{j^{\prime \prime }}^{s_{j^{\prime \prime }}}=g^{-z_{1}vk_{j^{\prime \prime }}r_{j^{\prime \prime }}\left(z_{2}-\sum_{j=1,j\neq j^{\prime \prime }}^{n}s_{j}\right)},\;{\tilde{v}}_{b,j^{\prime \prime }}={}^{\neg }v_{j^{\prime \prime }}\\ {\bar{u}}_{j^{\prime \prime }+n}^{s_{j^{\prime \prime }}}=g^{-z_{1}vk_{j^{\prime \prime }}r_{j^{\prime \prime }+n}\left(z_{2}-\sum_{j=1,j\neq j^{\prime \prime }}^{n}s_{j}\right)},\;{\tilde{v}}_{b,j^{\prime \prime }}=v_{j^{\prime \prime }} \end{array}\right.$$

When *j* ≠ *j*″, $\left[C_{j,1}^{*},C_{j,2}^{*}\right]$ in the ciphertext is computed as

$$C_{j,1}^{*}=X_{j}^{s_{j}}=g^{\frac{\beta s_{j}}{vk_{j}}}$$

$$C_{j,2}^{*}=u_{j}^{\prime }{}^{s_{j}}=\left\{ \begin{array}{l} u_{j}^{s_{j}}=g^{-vk_{j}r_{j}s_{j}},\;{\tilde{v}}_{b,j}=v_{j}\\ u_{j+n}^{s_{j}}=g^{-vk_{j}r_{j+n}s_{j}},\;{\tilde{v}}_{b,j}={}^{\neg }v_{j} \end{array}\right.$$

Finally, the simulator B sends $C{T^{\prime }}^{*}=\left(C^{*},C_{1}^{*},{\left\{\left(C_{j,1}^{*},C_{j,2}^{*}\right)\right\}}_{j\in \left[1,n\right]}\right)$ to adversary A.

**Phase 2.** The adversary A makes queries similarly to Phase 1 with the restriction that $\gamma \left(Attr,S_{0}^{*}\right)=0\Lambda \gamma \left(Attr,S_{1}^{*}\right)=0$.

**Guess.** The adversary A gives a guess *b*′ of *b*. If *b*′ = *b*, the simulator B outputs *τ* = 1, it demonstrates $Q=g^{z_{1}z_{2}}$. The adversary A exactly simulates the initial game, because

$$C^{*}=ck_{b}\cdot Y^{s}=ck_{b}\cdot e{\left(g,g\right)}^{\alpha s}=ck_{b}\cdot e\left(g,Q\right)=ck_{b}\cdot e{\left(g,g\right)}^{z_{1}z_{2}}$$

Otherwise the simulator B outputs *τ* = 1, it demonstrates that *Q* is a random element in G.

The advantage of adversary A to win the game is defined as

$$ADV\left(A\right)=\left|\mathrm{Pr}\left[b^{\prime }=b\right]-\frac{1}{2}\right|$$

Hence, if adversary A who is able to distinguish games *Game*_0_ and *Game*_1_ with a non-negligible advantage, simulator B is capable of breaking DDH assumption with a non-negligible advantage.

If ${\hat{v}}_{j}^{}$ belongs to $\left({\hat{v}}_{j}^{}\in S_{0}^{*}\Lambda {\hat{v}}_{j}^{}\in S_{1}^{*}\right)$ or $\left({\hat{v}}_{j}^{}\notin S_{0}^{*}\Lambda {\hat{v}}_{j}^{}\notin S_{1}^{*}\right)$, the ciphertext component {*C*_*j*,1_}_*j*∈[1,*n*]_ is the same as initial scheme, but for ${\hat{v}}_{j}^{}$ belongs to $\left({\hat{v}}_{j}^{}\in S_{0}^{*}\Lambda {\hat{v}}_{j}^{}\notin S_{1}^{*}\right)$ or $\left({\hat{v}}_{j}^{}\notin S_{0}^{*}\Lambda {\hat{v}}_{j}^{}\in S_{1}^{*}\right)$, the ciphertext component {*C*_*j*,1_}_*j*∈[1,*n*]_ will be replaced by random value {*Z*_*j*,1_}_*j*∈[1,*n*]_, which is represented as our pre-defined a series of games *Game*_2_,⋯,*Game*_*n*_,*Game*_*n*+1_. The DDH assumption is used to prove that *Game*_*l*_ and *Game*_*l*+1_ are indistinguishable, for *l*∈[1,*n*].

**Theorem 4.** If there is an adversary A who is able to distinguish games *Game*_*l*_ and *Game*_*l*+1_ with a non-negligible advantage, a simulator B is capable of breaking DDH assumption with a non-negligible advantage.

Proof: Supposing that a tuple $\left(g,g^{z_{1}},g^{z_{2}},Q\right)$ which is an instance of DDH problem, where g,Q∈G, $z_{1},z_{2}\in {\mathbb{Z}}_{p}^{*}$, is given to a simulator B, the simulator B performs the following operations:

**Initialization.** The adversary A submits two challenge access policies $S_{0}^{*}={\tilde{v}}_{0,1}{\tilde{v}}_{0,2}\cdots {\tilde{v}}_{0,n}$ and $S_{1}^{*}={\tilde{v}}_{1,1}{\tilde{v}}_{1,2}\cdots {\tilde{v}}_{1,n}$ to simulator B, then B throws a fair coin to select *b*∈{0,1}. When an attribute set $Attr={\hat{v}}_{1}{\hat{v}}_{2}\cdots {\hat{v}}_{n}$ satisfies that $\left({\hat{v}}_{j}^{}\in S_{0}^{*}\Lambda {\hat{v}}_{j}^{}\in S_{1}^{*}\right)$ or $\left({\hat{v}}_{j}^{}\notin S_{0}^{*}\Lambda {\hat{v}}_{j}^{}\notin S_{1}^{*}\right)$, *Game*_*l*_ and *Game*_*l*+1_ are the same according to game definition. Therefore, only the case of $\left({\hat{v}}_{j}^{}\in S_{0}^{*}\Lambda {\hat{v}}_{j}^{}\notin S_{1}^{*}\right)$ or $\left({\hat{v}}_{j}^{}\notin S_{0}^{*}\Lambda {\hat{v}}_{j}^{}\in S_{1}^{*}\right)$ is considered below.

**Setup.** The simulator B runs **Setup** algorithm, selects a bilinear map $e:G\times G\to G_{T}$, where $G,G_{T}$ are two multiplicative cyclic groups with prime order *p*, *g* is a generator of group G. It randomly chooses $\alpha ,\beta ,vk_{j}\in {\mathbb{Z}}_{p}$, sets *Y* = *e*(*g*,*g*,)^*α*^, it computes $X_{j}=g^{\frac{\beta }{vk_{j}}}=g^{\frac{z_{1}}{vk_{j}}}$, where $vk_{j}\in {\mathbb{Z}}_{p}\left(j\in \left[1,n\right]\right)$ as initial version number of attribute, implicitly lets *β* = *z*_1_. Afterwards, B picks up ${\left\{r_{i}\in {\mathbb{Z}}_{p}\right\}}_{i\in \left[1,2n\right]}$ at random, when ${\hat{v}}_{j}\in S_{b}^{*}$, there are $u_{j}=g^{-vk_{j}r_{j}}\left({\hat{v}}_{j}=v_{j}={\tilde{v}}_{j}\right)$ and $u_{j+n}=g^{-vk_{j}r_{j+n}}\left({\hat{v}}_{j}={}^{\neg }v_{j}={\tilde{v}}_{j}\right)$, when ${\hat{v}}_{j}\notin S_{b}^{*}$, there are ${\bar{u}}_{j}=g^{-z_{1}vk_{j}r_{j}}\left({\hat{v}}_{j}=v_{j}\neq {\tilde{v}}_{j}\right)$ and ${\bar{u}}_{j+n}=g^{-z_{1}vk_{j}r_{j+n}}\left({\hat{v}}_{j}={}^{\neg }v_{j}\neq {\tilde{v}}_{j}\right)$, where *j*∈[1,*n*]. The simulator B publishes public key $PK=\left(e,G,G_{T},g,Y,{\left\{\left(X_{j},u_{j},u_{j+n},{\bar{u}}_{j},{\bar{u}}_{j+n}\right)\right\}}_{j\in \left[1,n\right]}\right)$ and keeps master secret key *MSK* = (*α*,*β*,{*vk*_*j*_,}_*j*∈[1,*n*]_,{*r*_*i*_}_*i*∈[1,2,*n*]_) secret.

**Phase 1.** The adversary A commits a query attribute set $Attr={\hat{v}}_{1}{\hat{v}}_{2}\cdots {\hat{v}}_{n}$ to simulator B.

Secret key query: The simulator B randomly chooses $u,\lambda_{j}\in {\mathbb{Z}}_{p}$, *j*∈[1,*n*]. The local secret key *SK*_2_ is computed as $D_{1}=g^{\alpha +\beta u}=g^{\alpha +z_{1}u}$ and $D_{j,1}=X_{j}^{\lambda_{j}}=g^{\frac{\beta \lambda_{j}}{vk_{j}}}=g^{\frac{z_{1}\lambda_{j}}{vk_{j}}}$,when ${\hat{v}}_{j}=v_{j}$, B computes $D_{j,2}=g^{vk_{j}\left(u-r_{j}\lambda_{j}\right)}$, otherwise $D_{j,2}=g^{vk_{j}\left(u-r_{j+n}\lambda_{j}\right)}$, where *j*∈[1,*n*].

The simulator B sends *SK*_2_ = (*D*_1_,{(*D*_*j*,1_,*D*_*j*,2_)}_*j*∈[1,*n*]_ to adversary A.

**Challenge.** For access policies $S_{0}^{*}={\tilde{v}}_{0,1}{\tilde{v}}_{0.2}\cdots {\tilde{v}}_{0,n}$ and $S_{1}^{*}={\tilde{v}}_{1,1}{\tilde{v}}_{1,2}\cdots {\tilde{v}}_{1,n}$, the adversary A submits two equal-length keys *ck*_1_ and *ck*_2_. The simulator B executes **Encryption key-Encrypt** algorithm with $S_{b}^{*}$ and *ck*_*b*_, where *b*∈{0,1}. It sets $C_{1}^{*}=g^{s}=g^{z_{2}}$ and $C^{*}=ck_{b}\cdot Y^{s}=ck_{b}\cdot e{\left(g,g\right)}^{\alpha z_{2}}=ck_{b}\cdot e{\left(g,g^{z_{2}}\right)}^{\alpha }$, implicitly lets *s* = *z*_2_. Hereafter, for all *j*∈[1,*n*], the simulator B arbitrarily chooses $s_{j}\in {\mathbb{Z}}_{p}$ if *j* ≠ *n*, it computes $s_{n}=z_{2}-\sum_{j=1,j\neq n}^{n}s_{j}$ if *j* = *n*.

When *j* = *n*, $\left[C_{n,1}^{*},C_{n,2}^{*}\right]$ in the ciphertext is computed as

$$C_{n,1}^{*}=X_{n}^{s_{l}}=g^{\frac{z_{1}}{vk_{n}}\left(z_{2}-\sum_{j=1,j\neq n}^{n}s_{j}\right)}=Q^{\frac{1}{vk_{n}}}/g^{\frac{z_{1}}{vk_{n}}\sum_{j=1,j\neq n}^{n}s_{j}}$$

$$C_{n,2}^{*}=u_{n}^{\prime }{}^{s_{n}}=\left\{ \begin{array}{l} u_{n}^{s_{n}}=g^{-vk_{n}r_{n}\left(z_{2}-\sum_{j=1,j\neq n}^{n}s_{j}\right)},\;{\tilde{v}}_{b,n}=v_{n}\\ u_{2n}^{n}=g^{-vk_{n}r_{2n}\left(z_{2}-\sum_{j=1,j\neq n}^{n}s_{j}\right)},\;{\tilde{v}}_{b,n}={}^{\neg }v_{n} \end{array}\right.$$

When *j* ≠ *n*, $\left[C_{j,1}^{*},C_{j,2}^{*}\right]$ in the ciphertext is computed as

$$C_{j,1}^{*}=X_{j}^{s_{j}}=g^{\frac{\beta s_{j}}{vk_{j}}}$$

$$C_{j,2}^{*}=u_{j}^{\prime }{}^{s_{j}}=\left\{ \begin{array}{l} {\bar{u}}_{j}^{s_{j}}=g^{-z_{1}vk_{j}r_{j}s_{j}},\;{\tilde{v}}_{b,j}={}^{\neg }v_{j}\\ {\bar{u}}_{j+n}^{s_{j}}=g^{-z_{1}vk_{j}r_{j+n}s_{j}},\;{\tilde{v}}_{b,j}=v_{j} \end{array}\right.$$

Then the simulator B transmits $C{T^{\prime }}^{*}=\left(C^{*},C_{1}^{*},{\left\{\left(C_{j,1}^{*},C_{j,2}^{*}\right)\right\}}_{j\in \left[1,n\right]}\right)$ to adversary A.

**Phase 2.** The adversary A continues to query similarly to Phase 1 with the restriction that $\left({\hat{v}}_{j}^{}\in S_{0}^{*}\Lambda {\hat{v}}_{j}^{}\notin S_{1}^{*}\right)$ or $\left({\hat{v}}_{j}^{}\notin S_{0}^{*}\Lambda {\hat{v}}_{j}^{}\in S_{1}^{*}\right)$.

**Guess.** The adversary A gives a guess *b*′ of *b*. If *b*′ = *b*, the simulator B outputs *τ* = 1, it demonstrates $Q=g^{z_{1}z_{2}}$. The adversary A exactly simulates the initial game, because

$$C_{n,1}^{*}=X_{n}^{s_{l}}=g^{\frac{z_{1}}{vk_{n}}\left(z_{2}-\sum_{j=1,j\neq n}^{n}s_{j}\right)}=Q^{{}^{\frac{1}{vk_{n}}}}/g^{\frac{z_{1}}{vk_{n}}\sum_{j=1,j\neq n}^{n}s_{j}}={\left(g^{z_{1}z_{2}}\right)}^{\frac{1}{vk_{n}}}/g^{\frac{z_{1}}{vk_{n}}\sum_{j=1,j\neq n}^{n}s_{j}}$$

Otherwise the simulator B outputs *τ* = 0, it demonstrates that *Q* is a random element in G. The advantage of adversary A to win the game is defined as

$$ADV\left(A\right)=\left|\mathrm{Pr}\left[b^{\prime }=b\right]-\frac{1}{2}\right|$$

Therefore, if adversary A who is able to distinguish games *Game*_*l*_ and *Game*_*l*+1_ with a non-negligible advantage, simulator B is capable of breaking DDH assumption with a non-negligible advantage.

In a word, if for all polynomial-time adversary who could win the game with a negligible advantage, our scheme will achieve selective ciphertext security under chosen-plaintext attacks.

### 5.4 Forward and backward security

A data user's attribute set satisfies access structure, only when the search keyword set is included in the encrypted keyword set, the target ciphertext can be searched. However, an attribute *attr*_*j*′_ of data user needs to be revoked at a certain time, the system will inform all non-revoked data users to update secret keys that associated with revoked attribute *attr*_*j*′_ and the cloud server completes ciphertexts update. During the initial phase, we select a randomized version number for each attribute. As long as update occurs, the version number of attribute will be changed. Afterwards, an update key *UK*_*j*′_ will be used to re-encrypt ciphertexts, and update secret keys relevant to revoked attribute of all non-revoked data users, the data user cannot continue performing keyword search and decryption with previous token and secret key. Because in the search phase, the updated keyword index cannot match data user's original token, namely

$$\frac{e\left({\tilde{u}}^{*},v^{z}\right)e\left({\tilde{\sigma }}^{z},g\right)}{{\tilde{y}}^{z}}\neq e{\left(g,g\right)}^{t_{2}acz}\;\left(vk_{j^{\prime }}^{*}\neq vk_{j^{\prime }}\right)$$

During the decryption phase, the data user's original secret key cannot decrypt updated ciphertext, that is

$$\frac{C\prod_{j=1,j\neq j^{\prime }}^{n}e\left(C_{j,1}^{},D_{j,2}\right)e\left(C_{j^{\prime },1}^{*},D_{j^{\prime },2}\right)}{e\left(C_{1},D_{1}\right)\prod_{j=1,j\neq j^{\prime }}^{n}e\left(C_{j,2}^{},D_{j,1}\right)e\left(C_{j^{\prime },2}^{*},D_{j^{\prime },1}\right)}\neq ck\;\left(vk_{j^{\prime }}^{*}\neq vk_{j^{\prime }}\right)$$

Therefore, the attribute revocation in our scheme achieves backward security.

A data user's attribute set satisfies access structure, when an attribute *attr*_*j*′_ of data user needs to be revoked, the version number *vk*_*j*′_ corresponding to attribute will be changed, namely it will be randomized by a random value $vk_{j^{\prime }}^{*}$. Hereafter, the system will inform update secret keys that related to revoked attribute of all non-revoked data users, updated secret keys and tokens are returned to effective data users. Simultaneously, the partial ciphertexts are relevant to revoked attribute are also updated. Even if the data user saves the ciphertext before joining system, because of $vk_{j^{\prime }}^{*}\neq vk_{j^{\prime }}^{}$, the previous keyword cannot be searched with the updated token and the previous ciphertext cannot be decrypted with the updated secret key. Therefore, the attribute revocation in our scheme achieves forward security.

## Performance and efficiency analyses

This section mainly analyzes performance and efficiency comparisons between our scheme and literature [31–35]. The performance comparison is to compare functional differences between our scheme and other schemes. The efficiency comparison is to analyze operation time differences both our proposal and other schemes in a certain phase. Based on Pairing Cryptography (PBC) library [36] and group operation with prime order *p*, we mainly consider three kinds of operations on time complexity: exponential operation, multiplication operation and pair operation. Specifically, *K* represents the number of attributes that satisfy access structure and *N* represents the number of attributes owned by data user. (In our scheme, *K* = *N*)

Table 1 shows performance comparison between our scheme and literature [31–35]. The literature [31] supports multi-keyword search and security proof under the hard problem, but it does not support attribute-based encryption and revocation. The literature [32–35] all support attribute-based encryption, meanwhile literature [35] also supports direct revocation, while our scheme supports attribute revocation. In addition, the literature [32] does not support security proof under the hard problem. Compared with other solutions in the Table 1, our proposal supports all functions.

**Table 1:** Performance comparison.

Scheme	Multi- keyword search	Attribute-based encryption	revocation	Security proof under the hard problem
*[31]*	√	×	×	√
*[32]*	×	√	×	×
*[33]*	×	√	×	√
*[34]*	×	√	×	√
*[35]*	×	√	√	√
*Ours*	√	√	√	√

×: The scheme does not support corresponding function

√: The scheme supports corresponding function

Table 2 shows efficiency comparison between the proposed scheme and the literature [31–35]. Since literature [32–35] do not support multi-keyword search, the proposed scheme and literature [31] have requirements for the number of keywords in the encryption, token generation, and search phases. For comparison, we consider that the quantity of encrypted keywords is equivalent to the quantity of search keywords, that is *r* = *d*.

**Table 2:** Efficiency comparison.

Scheme	Encryption	Token generation	Search	Decryption
*[31]*	(2*K*+2*r*+1)*E*+(*K*+*r*)*M*′+(2*r*+1)*E*_*T*_	(4*N*+2*d*)*E*+(2*N*+*d*)*M*′	(2*N*+*d*)*P*+*dE*_*T*_+*M*_*T*_	—
*[32]*	*KP*+(4*K*+1)*E*+*KM*′+(2*K*+1)*E*_*T*_+*KM*_*T*_	—	—	3*NP*+(3*N*+1)*M*_*T*_
*[33]*	(2*K*+3)*E*+(*K*+1)*M*′+*E*_*T*_	(4*N*+5)*E*+(2*N*+2)*M*′	3*P*+*E*_*T*_+2*M*_*T*_	(2*N*+2)*P*+(*N*+1)*M*′+2*NE*_*T*_+3*M*_*T*_
*[34]*	(8*K*+3)*E*+*E*_*T*_+(2*K*+1)*M*′	—	—	2*E*+*M*′+*NE*_*T*_+(5*N*+1)*M*_*T*_+(6*N*+1)*P*
*[35]*	(4*K*+2)*E*+(3*K*−1)*M*′+*E*_*T*_	—	—	(*N*−1)*E*+(*N*−2)*M*′+(*N*+1)*E*_*T*_+(2*N*+4)*M*_*T*_+(2*N*+4)*P*
*Ours*	(2*K*+*r*+4)*E*+(*K*+*r*)*M*′+*E*_*T*_	(2*N*+*d*+4)*E*+(*N*+*d*)*M*′+*E*_*T*_+*NM*_*T*_	(*d*+3)*P*+(*d*+3)*M*_*T*_	(2*N*+1)*P*+(2*N*+1)*M*_*T*_

*E*: An exponential operation of elements in group G

*E*_*T*_: An exponential operation of elements in group $G_{T}$

*M*′: A multiplication operation on group G

*M*_*T*_: A multiplication operation on group $G_{T}$

*P*: A pair operation

—: The scheme does not include this calculation

Through experiments on common cryptographic algorithms, the operation time of each phase is obtained and the following operation time comparison chart is drawn. The environment of the hardware runtime is Intel Core i5-3470 CPU @ 3.20GHz, and RAM is 4.00GB. The software runtime environment is JDK 1.7.5, JPBC 2.0.0 and MyEclipse 10.

**Fig 2:**
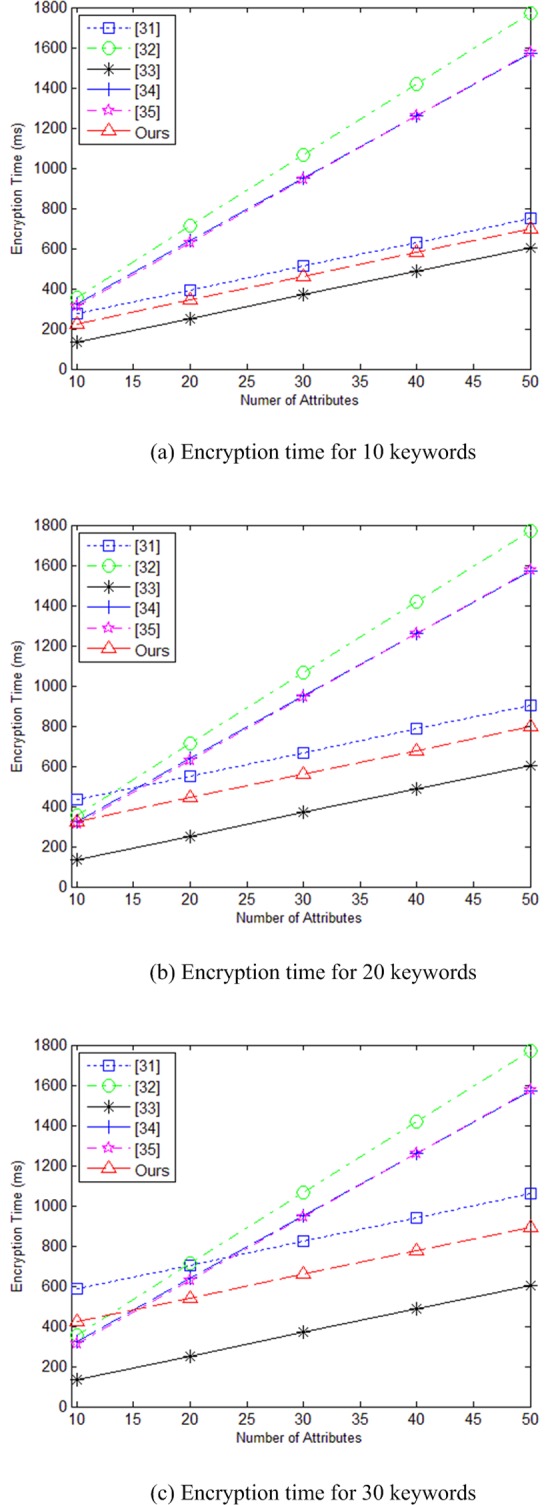
(a) Encryption time for 10 keywords (b) Encryption time for 20 keywords. (c) Encryption time for 30 keywords.

In Fig 2(A), 2(B) and 2(C) are time charts for running encryption algorithm as the number of attributes increases when keyword set contains 10, 20, and 30 keywords, respectively. Since literature [32–35] do not support multi-keyword search function, the proposed scheme is mainly compared with literature [31]. From the Fig 2, we can see that in the encryption phase, the calculation speed of our scheme is slower than literature [33], faster than literature [31,32,34,35].

**Fig 3:**
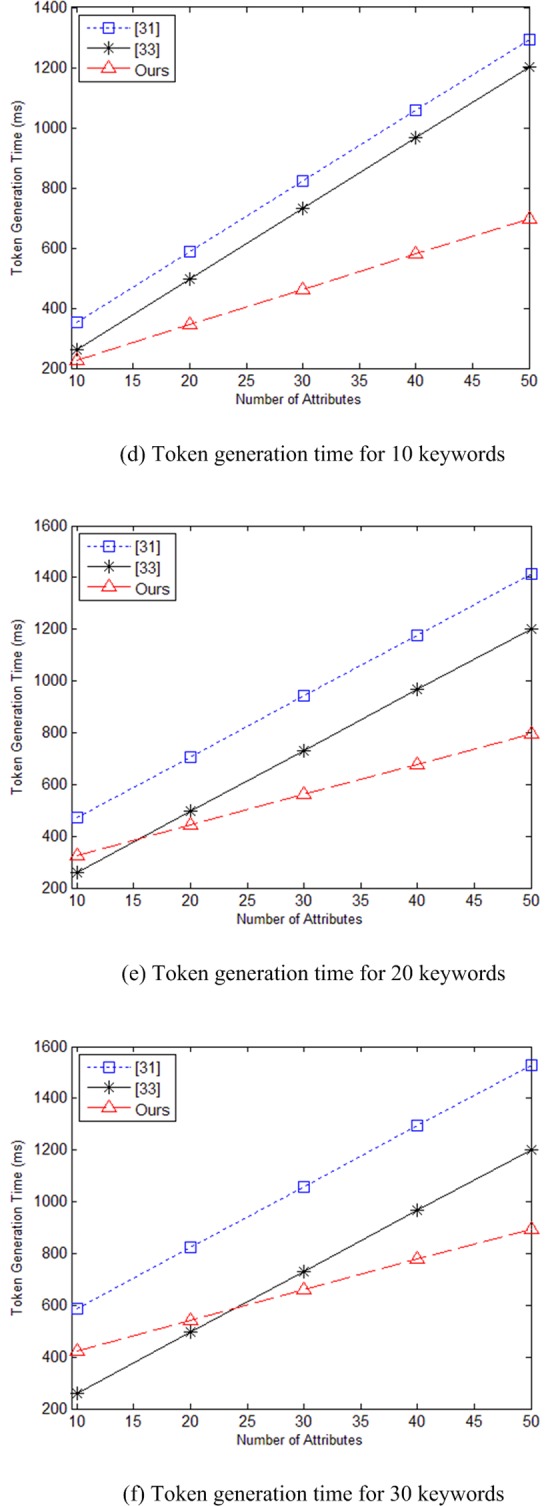
(d) Token generation time for 10 keywords (e) Token generation time for 20 keywords (f) Token generation time for 30 keywords.

In Fig 3(D), 3(E) and 3(F) are time charts for running token generation algorithm as the number of attributes increases when keyword set contains 10, 20, and 30 keywords, respectively. Since literature [32,34,35] does not involve token generation algorithm, at this phase, our scheme is mainly compared with literature [31,33], and because literature [33] does not support multi-keyword search function, the changes in the number of keywords will only lead to changes in the algorithm time of our scheme and literature [31]. As can be seen from the Fig 3, when the number of keywords is 10, the calculation time of our scheme is shortest. When the number of keywords increases and the number of attributes is small, the computation speed of the proposed scheme is slower than literature [33]. However, as the number of attributes increases, the advantage of our scheme become more significant, and the algorithm speed is superior to literature [31,33].

**Fig 4:**
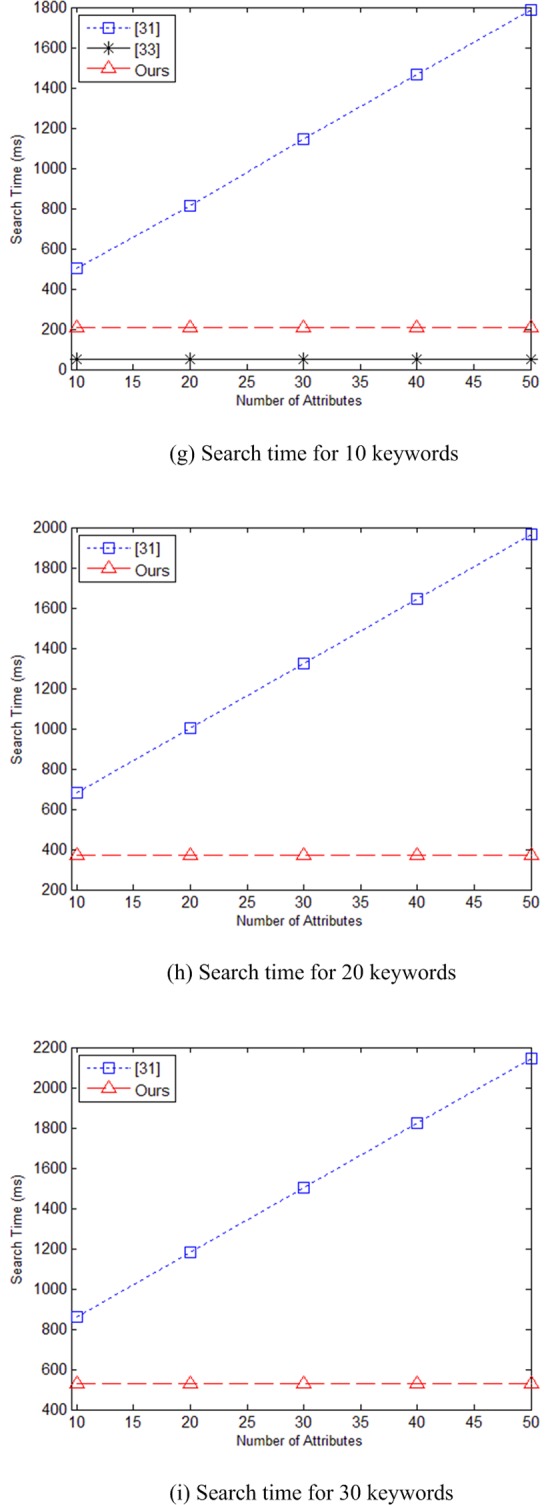
(g) Search time for 10 keywords (h) Search time for 20 keywords (i) Search time for 30 keywords.

In Fig 4(G), 4(H) and 4(I) are the time charts for running search algorithm as the number of attributes increases when keyword set contains 10, 20, and 30 keywords, respectively. Since literature [33] does not involve the number of attributes and keywords in the search phase, the algorithm time in [33] is constant. We have focused on comparing our scheme with literature [31], Fig 4(H) and Fig 4(I) no longer calculate the computation time of literature [33]. Since our scheme do not involve the number of attributes in the search phase, the algorithm time of our scheme is only associated with the number of keywords. From the Fig 4, we can see that at this phase, the calculation speed of the proposed scheme is faster than literature [31].

**Fig 5:**
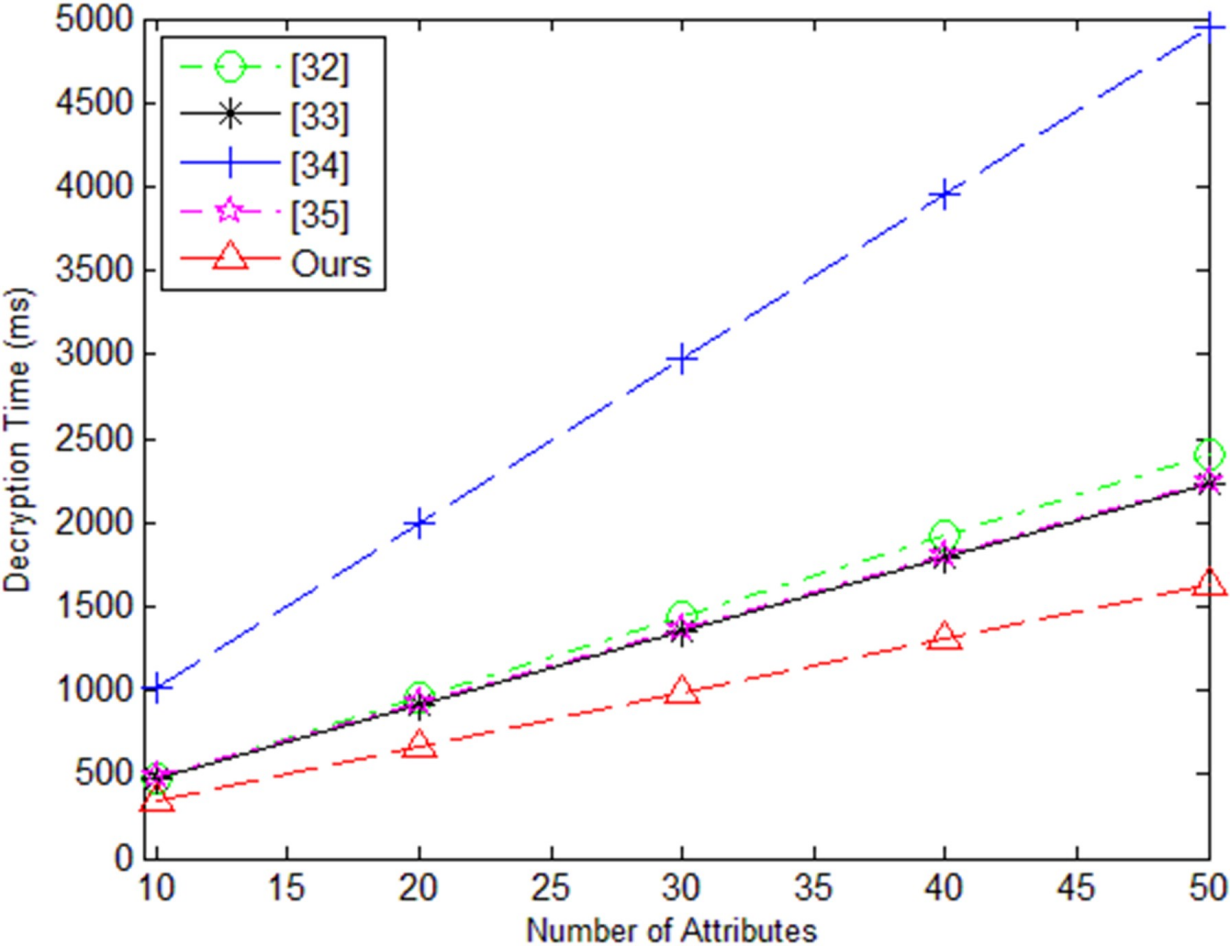
Decryption time.

The Fig 5 is the algorithm time figure in the decryption phase of our scheme and literature [32–35]. It can be seen from the Fig 5 that with continuous increase of the quantity of attributes, calculation time of our solution in this phase is less than literature [32–35].

## Conclusion

We propose an attribute-based encryption scheme with multi-keyword search and supporting attribute revocation in cloud storage. On the one hand, based on the traditional CP-ABE schemes, our proposal uses the AND-gate access policy, adds attribute revocation and multi-keyword search, which is more practical. On the other hand, we present security proof under the complexity assumption, it demonstrates that our scheme is secure. Finally, we analyze performance and efficiency of proposed scheme and other solutions via experimental evaluation, which indicates that our scheme is more efficient.

In the future, how to achieve a dynamic search with multiple functions and direct revocation will be a project that needs further study.

## Supporting information

S1 File The runtime of cryptographic operations. (DOCX)

S2 File Notation definition. (DOCX)
