# Supplementary material for: Attribute-based encryption scheme with multi-keyword search and supporting attribute revocation in cloud storage
Source: PLoS One. 2018 Oct 12;13(10):e0205675. doi: 10.1371/journal.pone.0205675 (PMC6185864; doi:10.1371/journal.pone.0205675)
Supplement: S2 File — (DOCX) [file pone.0205675.s002.docx]

**S2 File**

The symbols proposed in our paper are defined as follows:

**Notation definition**

| **Symbol** | **Descriptions** |
| --- | --- |
| $U=\left\{attr_{1},attr_{2},\cdots,attr_{n}\right\}$ | Universe set of attributes |
| $Attr=\hat{v}_{1}\hat{v}_{2}\cdots\hat{v}_{n}$ | Attribute set of data user |
| $\hat{v}_{j}$ | The value of *j*-th attribute in data user's attribute set |
| $S=\tilde{v}_{1}\tilde{v}_{2}\cdots\tilde{v}_{n}$ | Access policy |
| $\tilde{v}_{j}$ | The value of *j*-th attribute in access policy |
| $vk_{j}$ | The version number of *j*-th attribute |
| $WD=\left\{w_{1},w_{2},\cdots w_{r}\right\}$ | Encrypted keyword set |
| $WD'=\left\{w'_{1},w'_{2},\cdots,w'_{d}\right\}$ | Searched keyword set |
| $\Pi$ | Keyword space |
| $Index$ | Index of keyword set $WD$ |
| $CT'$ | Ciphertext of encryption key |
| $Cph$ | The overall ciphertext |
| $Tok$ | Token of keyword set $WD'$ |
